# Interactions Between Marine Group II Archaea and Phytoplankton Revealed by Population Correlations in the Northern Coast of South China Sea

**DOI:** 10.3389/fmicb.2021.785532

**Published:** 2022-01-25

**Authors:** Songze Chen, Jianchang Tao, Yufei Chen, Wenxiu Wang, Lu Fan, Chuanlun Zhang

**Affiliations:** ^1^Department of Ocean Science and Engineering, Southern University of Science and Technology, Shenzhen, China; ^2^Shenzhen Key Laboratory of Marine Archaea Geo-Omics, Shenzhen, China; ^3^Southern Marine Science and Engineering Guangdong Laboratory (Guangzhou), Guangzhou, China; ^4^Shanghai Sheshan National Geophysical Observatory, Shanghai, China

**Keywords:** MGII archaea, phytoplankton, South China Sea, coastal surface water, network

## Abstract

Marine Group II (MGII) archaea (Poseidoniales) are the most abundant surface marine planktonic archaea and are widely distributed in both coastal and pelagic waters. The factors affecting their distribution and activity are poorly understood. MGII archaea have the metabolic potential to utilize algae-derived organic matter and are frequently observed in high abundance during or following phytoplankton blooms, suggesting that they are key players of the marine food web. In this study, we studied interactions between MGII archaea and the diverse taxa of phytoplankton in the northern coast of South China Sea. Non-metric multidimensional scaling and cluster analyses demonstrated distinct MGII community patterns in the Pearl River plume (PRP) and the open regions of the northern South China Sea (ONSCS), with MGIIb dominating the former and MGIIa and MGIIb showing remarkable variations in the latter for the same sampling season. Nevertheless, positive correlations (Pearson correlation: *R* > 0.8 and *P* < 0.01) in absolute abundances of ribosomal RNA (rRNA)-derived complementary DNA and rRNA genes from network analyses were found between MGII archaea and phytoplankton (cyanobacteria, haptophytes, and stramenopiles in both PRP and ONSCS) among different particle size fractions, indicating their intrinsic relationships under changing environmental conditions. The results of this study may shed light on the multiple interactions between co-existing species in the micro-niches of different oceanic regions.

## Introduction

Planktonic archaea are one of the fundamental life forms in the ocean and play essential roles in ecological function and biogeochemical cycles. They amount up to ∼1.3 × 10^28^ cells, in the same order of magnitude as the bacteria in seawater (Karner et al., 2001). The major archaeal group Marine Group II (MGII) is widely distributed in the global ocean (Massana et al., 2000; Wendeberg et al., 2002; Zhang et al., 2015) and has relatively higher biomass in the surface waters than in the deep waters of the open ocean (Karner et al., 2001; Wendeberg et al., 2002; Alderkamp et al., 2006; Lincoln et al., 2014; Liu et al., 2017; Xie et al., 2018; Santoro et al., 2019). MGII archaea are recently named *Candidatus* Poseidoniales at the order level, which comprises two major family-level groups, namely, MGIIa (*Candidatus* Poseidonaceae) and MGIIb [*Candidatus* Thalassarchaeaceae, previously named as Thalassarchaea (Martin-Cuadrado et al., 2015)], each comprising of numerous ecologically and metabolically diverse subclades or genera (Rinke et al., 2019; Tully, 2019).

Aquatic phytoplankton is one of the most important co-existing microorganisms with MGII archaea; the latter are presumed to consume the organic substrates released by the former (Stoica and Herndl, 2007; Hugoni et al., 2013; Lima-Mendez et al., 2015). MGII were observed to co-exist with a variety of phytoplankton such as dinophyta, chlorophyta, bacillariophyta, and cyanobacteria in the Pearl River mouth (Xie et al., 2018; Wang et al., 2019). They were also found to co-occur with green algae (chlorophyta)-*Micromonas pusilla* and *Bathycoccus*, haptophyta-*Phaeocystis*, and cryptophyta-*Teleaulax* or in a delayed phase with diatom-*Chaetoceros* and rhaphidophyta-*Heterosigma* in other coastal waters (Orsi et al., 2015; Needham and Fuhrman, 2016; Needham et al., 2018; Orellana et al., 2019). All MGIIa and nearly half of MGIIb genomes possess the archaeal flagella gene operon. It is suggested that flagella are used by these archaea to attach to phytoplankton cells (Lassak et al., 2012; Rinke et al., 2019). Chitinase, glycoside hydrolase, and protease expressed by MGII archaea may have the function of cracking high-molecular-weight organic matters such as oligosaccharide agarose or agaropectin from intact phytoplankton biomass (Martin-Cuadrado et al., 2015; Zhang et al., 2015; Orsi et al., 2016; Rinke et al., 2019; Tully, 2019; Damashek et al., 2021).

Most of the above-mentioned co-occurrence studies were based on the relative abundances of MGII archaea and phytoplankton. It is unclear how the diverse subgroups of MGII archaea interact with specific phytoplankton types in the coastal waters (Galand et al., 2010; Orsi et al., 2015; Xie et al., 2018), and thus the potential phytoplankton control on the distribution of MGII archaeal populations in coastal waters is still elusive.

The ribosomal RNA (rRNA) gene has been proven to be effective for characterizing the phylogenetic and taxonomic structure of microbial assemblages, and the rRNA has been widely applied to characterize active microbes in a mixed community (Blazewicz et al., 2013; Hugoni et al., 2013). Here, we used phytoplankton 23S rRNA gene primers and newly designed MGII-specific 16S rRNA gene primers to quantify rRNA gene/rRNA-derived complementary DNA (cDNA) abundances of phytoplankton (including both cyanobacteria and chloroplasts of microbial eukaryotes) taxa and MGII archaea subgroups, respectively, in the Pearl River plume (PRP) and the open regions of the northern South China Sea (ONSCS). Quantitative real-time PCR (qPCR) and sequencing were performed on both DNA and RNA samples. Features of the free-living (FL) and the particle-associated (PA) subcommunities were compared. We applied network analysis to explore the potential effect of the phytoplankton population on MGII archaea distribution and niche speciation.

## Materials and Methods

### Seawater Sample Collection and Physicochemical Analyses

**FIGURE 1:**
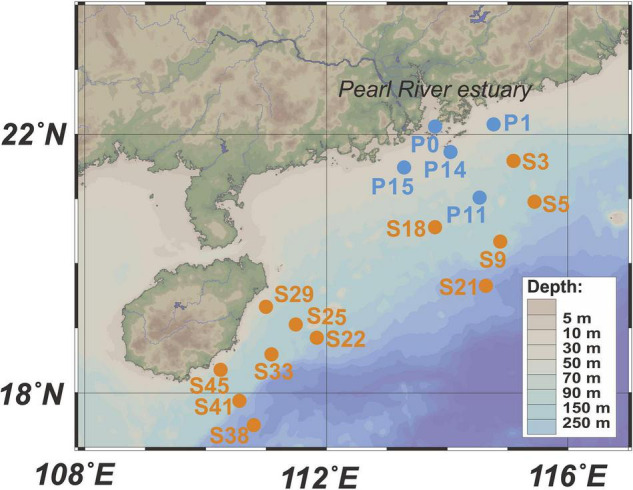
Locations of sampling stations. Blue dots represent stations in the PRP, and orange dots are stations in the ONSCS. Only surface water samples were analyzed in this study.

Surface seawater (5 m) sampling and hydrographic profiling (water depth, temperature, and salinity) were conducted in the PRP and ONSCS areas (Figure 1) using a conductivity–temperature–depth (CTD) rosette sampler (model SBE 9-11 Plus; Sea-Bird Electronics, Inc., Bellevue, WA, United States) equipped with 12-L Niskin sample bottles.

Around 2–4 L seawater was filtered with a vacuum of at most 100 Mbar through 2.7 μm (Glass Microfiber Membrane, Whatman 1823-047) and then 0.22 μm (Nitrocellulose Membrane, Millipore GSWP04700) pore-sized membrane filters to collect PA and FL microorganisms, respectively. Filters were preserved within 20 min of collection in liquid nitrogen till nucleic acid extraction. Filtrates were transferred into 50 ml centrifuge tubes and preserved at −20°C for nutrient analysis.

The concentration of NH_4_^+^ was analyzed by the indophenol blue spectrophotometric method (Sims et al., 1995); the NO_3_^–^, NO_2_^–^, PO_4_^3–^, and SiO_3_^2–^ were measured with a four-channel continuous-flow Technicon AutoAnalyzer 3 (AA3; Bran-Lube GmbH, Norderstedt, Germany).

### Nucleic Acid Extraction and Complementary DNA Generation

The filtered membranes for simultaneous extraction and purification of DNA and RNA follow the method introduced by Tournier et al. (2015) using the Fast DNA SPIN Kit for Soil (MP Biomedicals, Solon, OH, United States). Briefly, filtered membranes were cut into pieces to a Lysing Matrix E tube and frozen overnight at −80°C. A sodium phosphate buffer and MT buffer were then applied to lysate cells frozen in the tube. Cellular proteins were removed using a protein precipitation solution. The remaining DNA/RNA were collected using a binding matrix suspension and spin™ filter (total DNA can be diluted with DNase/pyrogen-free water). The filtrate (containing RNA) of spin™ filter was removed and transferred to a new centrifuge tube for further RNA purification. This was done by adding isopropanol and sodium acetate to precipitate RNA from the solution. The RNA precipitant was washed with 70% ethanol and resuspended in 100 μl of RNase-free water for storage (Tournier et al., 2015).

The raw RNA was subjected to DNA digestion by Recombinant DNase I (RNase-free, Takara, 2270B) and further purified by using the RNeasy MinElute Cleanup Kit (Qiagen, 74204). The residual DNA in purified RNA was quantified by qPCR analysis with primers Bac331F: TCCTACGGGAGGCAGCAGT and Bac797R: GGACTACCAGGGTATCTAATCCTGTT (Nadkarni et al., 2002) and was below the detection limit. cDNA was synthesized by using the PrimeScript™ II 1st Strand cDNA Synthesis Kit (TaKaRa, 6210A) with random hexamer primers.

### Microbial Ribosomal RNA and Ribosomal RNA Gene Quantification

QPCR analyses were performed on a QuantStudio5 (Thermo Fisher Scientific, Inc., Waltham, MA, United States) by using the QuantStudio5™ Design and Analysis Software v1.5.1. The primers targeting phytoplankton 23S rRNA genes were A23SrVF1: 5′-GGACARAAAGACCCTATG-3′ and A23SrVR1: 5′-AGATCAGCCTGTTATCC-3′ (Sherwood and Presting, 2007). The newly designed primers Ar-559F: THTTATTGGGCCTAAAACGTCCG and MGII-771R: TATCTA ATCCGGTTCGTGCCCCT were used to target MGII archaeal 16S rRNA genes (see Supplementary Material). The qPCR reaction volume was 10 μl containing 5 μl of TB Green™ Premix Ex Taq™ II (Takara, PR820A), 0.4 μM of each primer, and 1 μl of template DNA/cDNA. Thermal cycling consisted of initial denaturation at 95°C for 30 s, followed by 40 cycles of denaturation at 95°C for 5 s, annealing at 55°C for 45 s, and extension at 72°C for 60 s. QPCR was conducted in triplicates for each sample. The quantification standard was generated with a series of 10-fold diluted purified plasmid DNA (10^3^–10^8^ copies/μl) with cloned rRNA genes, which were amplified from a collection of the PRP and ONSCS water samples (Figure 1) by using primers A23SrVF1/A23SrVR1 and Ar-559F/MGII-771R, respectively (Sherwood and Presting, 2007). The *R*^2^ values of the qPCR standard curves were greater than 0.99, and the efficiency was 90–100%. QPCR results were discarded when the melt curves showed an evidence of primer dimers. Finally, we normalized the number of the rRNA (cDNA)/rRNA gene to copies per liter.

### Sequencing and Taxonomic Classification

High-throughput amplicon sequencing targeting the phytoplankton 23S rRNA and archaeal 16S rRNA genes was conducted to investigate the proportional changes of phytoplankton taxa and MGII archaea in archaeal communities, respectively. Specifically, a nest-PCR approach using primers A23SrVF1 and A23SrVR1 for the first round and the primers A23SrVF2: 5′-CARAAAGACCCTATGMAGCT-3′ and A23SrVR2: 5′-TCAGCCTGTTATCCCTAG-3′ for the second round (Sherwood and Presting, 2007; Yoon et al., 2016) was performed to amplify phytoplankton plastid 23S rRNA genes from both DNA and cDNA samples. Archaeal V4–V5 16S rRNA genes were amplified by using primers 524F10extF: 5′-TGYCAGCCGCCGCGGTAA-3′ and Arch958RmodR: 5′-YCCGGCGTTGAVTCCAATT-3′ (Pires et al., 2012). Sequencing was conducted by Majorbio Bio-Pharm Technology Co., Ltd. (Shanghai, China) according to the standard protocols of the Illumina MiSeq platform.

The raw sequences with average quality scores lower than 20 were filtered, and primer sequences were cut off by using USEARCH11 (Edgar, 2010). The resulting sequences of 200 bases and longer were dereplicated and denoised by chimera filtering, and zero-radius operational taxonomic units (ZOTUs) were obtained. The taxonomies of representative ZOTUs for the 16S rRNA gene were assigned against the Silva 138.1 database by using the RDP classifier implemented in QIIME v1.9.1 with a bootstrap cutoff of 80% (Caporaso et al., 2010; Quast et al., 2013). The ZOTU representatives of the MGII 16S rRNA gene were selected in TBtools (Chen et al., 2020) and taxonomically classified by searching against a custom database (Supplementary Table 1) by using BLASTn (similarity > 95% and *E*-value < 10^––5^; NCBI-blast-2.10.0+^1^). Phytoplankton taxonomies as Class/Order names were assigned to the 23S rRNA gene ZOTUs by using BLASTn (similarity > 90% and *E* < 10^–5^) against the Silva 138.1 LSU database because the annotation of most 23S rRNA sequences is not accurate enough at the genus level (Altschul et al., 1990). The accuracy of taxonomic classification was verified to the neighbor-joining phylogenetic tree in MEGA7 (Kumar et al., 2016; Supplementary Figure 2).

### Statistical and Ecological Analyses

The ZOTU sequence numbers were normalized to an equal number by subsampling in QIIME for a later statistical and ecological analysis (Caporaso et al., 2010). The alpha diversity of each sample was calculated by using Mothur-1.35.1 (Schloss et al., 2009). Non-metric multidimensional scaling (NMDS) was carried out to delineate community structure differences among samples based on the Bray–Curtis similarity matrix at the ZOTU level. The phytoplankton community structure differences affecting the MGII community was interpreted in the Mantel test based on the Bray–Curtis similarity matrix (Pearson, permutation = 999) at the ZOTU level by using the “vegan” R package (Oksanen et al., 2013).

The responses of the relative abundance of MGII archaeal and phytoplankton ZOTUs to the PRP and ONSCS areas were further determined using the linear discriminant analysis effect size (LEfSe) method^2^ with default parameters (Segata et al., 2011). “envfit” was used to compare and interpret the effects of environmental factors on the phytoplankton and MGII compositions with the Monte Carlo permutation test (permutation = 999) by using the “vegan” R package (Dixon, 2003).

iCAMP analysis was performed to unify niche and neutral perspectives on governing community structure based on MGII ZOTU relative abundances, phylogenetic tree, and environmental factors (Ning et al., 2020). The cluster analysis of phytoplankton and MGII genera proportions was performed by using TBtools (Chen et al., 2020).

The absolute abundances of phytoplankton or MGII genera/ZOTUs were calculated from each genus/ZOTU proportion multiplied by the total qPCR abundances in phytoplankton and MGII, respectively. Outlier tests were analyzed in LOF before doing a regression analysis of the total qPCR abundances (Breunig et al., 2000). Pearson correlations in phytoplankton and MGII ZOTUs’ absolute abundances and environmental factors were analyzed in RStudio (RStudio Team, 2020). The rRNA/rRNA gene abundances of MGII and phytoplankton were defined as the absolute abundances retrieved from cDNA/DNA.

The top 40 MGII ZOTUs of highest relative abundance and present in over half of the sequencing samples were selected for network analysis. The Pearson correlations (*R*^2^ > 0.64 and *P* < 0.01) of phytoplankton and MGII ZOTUs’ absolute abundances and environmental factors were visualized in Cytoscape 3.2.1 (Shannon et al., 2003). The hub ZOTUs of networks were identified based on the degree using CytoHubba (Chin et al., 2014).

### Nucleotide Sequence Accession Numbers

The raw reads generated in this project have been deposited at NCBI under the umbrella project number PRJNA748026.

## Results

### Physicochemical Characteristics

The water depths of the sites ranged from 15 to 86 m in the PRP and ranged from 37 to 200 m in the ONSCS except for two deep ocean sites S21 (1307 m) and S38 (1604 m). The temperature varied from 26.9 to 27.7°C in the PRP surface water and from 27.3 to 30.2°C in the ONSCS surface water (Supplementary Table 5). Except that NH_4_^+^ (0.0–1.8 μmol/L) was low in all samples, the concentrations of other nutrients were significantly (unpaired *t*-test: *P* < 0.001) higher in the PRP region (NO_3_^–^: 1.05–1.44 μmol/L, NO_2_^–^: 0.6–1.02 μmol/L, PO_4_^3–^: 0.04–1.17 μmol/L, and SiO_3_^2–^: 2.70–6.91 μmol/L) than that in the ONSCS region (NO_3_^–^: 0.45–0.87 μmol/L, NO_2_^–^: 0.00–0.11 μmol/L, PO_4_^3–^: 0.00–0.19 μmol/L, and SiO_3_^2–^: 1.04–4.62 μmol/L) (Supplementary Table 5 and Supplementary Figure 3).

### Abundances of Phytoplankton and Marine Group II Archaea Ribosomal RNA Sequences

In all samples, the phytoplankton rRNA gene and rRNA (cDNA) absolute abundances were 0.9–3.0 orders of magnitude higher than those of MGII archaea. Significantly higher abundances of phytoplankton and MGII were associated with the FL fractions than the PA fractions (paired one-tailed *t*-test: *P* < 0.01), except for the phytoplankton rRNA gene in the PRP (Supplementary Figure 4 and Supplementary Table 6). Both phytoplankton [one-way analysis of variance (ANOVA): *P* < 0.01] and MGII rRNA genes in PA fractions (one-way ANOVA: *P* < 0.01) had significantly higher abundance in the PRP than in the ONSCS, while in FL fractions, similar abundances existed between the two geographical locations. The abundances of both phytoplankton and MGII rRNA were significantly (*t*-test: *P* < 0.01) higher than that of the rRNA genes in FL fractions, but no significant difference was found in PA fractions.

Significant positive correlations were found between MGII and phytoplankton abundances in the PA-rRNA, PA-rRNA (cDNA), and FL-rRNA gene samples (*P* < 0.01, *R*^2^ > 0.64; Supplementary Figures 5A–C). The positive relationships between MGII and phytoplankton abundances in FL-RNA gene samples were less obvious, which may be due to the narrow distribution of abundances between the samples for both MGII archaea and phytoplankton (Supplementary Figures 5B,D).

### Community Structures and Potential Activities of Phytoplankton and Marine Group II Archaea

**FIGURE 2:**
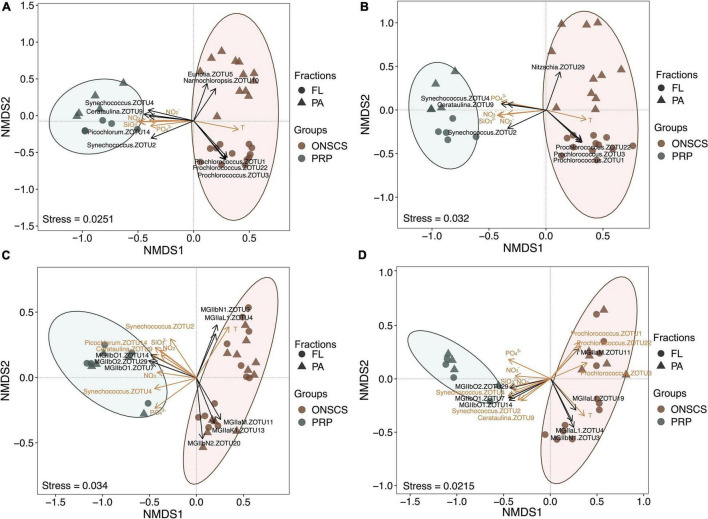
Non-metric multidimensional scaling analysis of the phytoplankton-rRNA gene **(A)**, phytoplankton-rRNA (cDNA, **B**), MGII-rRNA gene **(C)**, and MGII-rRNA (cDNA, **D**) based on the Bray–Curtis similarity matrix at the ZOTU (100% similarity) level. Sample grouping is based on the PRP and ONSCS areas. Significant differences (*P* < 0.05) of MGII archaeal and phytoplankton ZOTUs were added to NMDS based on the LEfSe method between the PRP and ONSCS areas (black arrows). ZOTUs and environmental factors that may significantly (*P* < 0.05) affect phytoplankton and MGII community structures were added as yellow arrows using the “envfit” function.

High-throughput amplicon sequencing targeting the phytoplankton and archaeal rRNA genes using the Illumina MiSeq platform was conducted to investigate the proportional changes of phytoplankton and MGII in archaeal communities. A total of 1,966,449 high-quality archaeal 16S rRNA sequences from 34 DNA and 28 RNA samples and 532,756 high-quality phytoplankton 23S rRNA sequences from 34 DNA and 33 RNA samples were obtained (Supplementary Tables 7, 8). Coverage estimation suggests that most sequence diversity in samples was captured (>97.8%). No significant differences of the MGII Shannon index were found in PRP-PA, PRP-FL, ONSCS-PA, and ONSCS-FL fractions for both rRNA genes and rRNA (Supplementary Table 8). The NMDS tests were carried out to delineate any ZOTU differences among sequencing samples (Figure 2).

The samples of the PRP and ONSCS can be well distinguished based on MGII and phytoplankton ZOTUs compositions. Multiple ZOTUs had similar trends in rRNA gene and rRNA samples, such as *Synechococcus*-ZOTU2, ZOTU4, *Cerataulina*-ZOTU9, MGIIb-O1-ZOTU7, -ZOTU14, and MGIIb-O2-ZOTU29, which were more abundant in the PRP than in the ONSCS. *Prochlorococcus*-ZOTU1, -ZOTU3, -ZOTU22, MGIIa-M-ZOTU11, MGIIb-N1-ZOTU3, and MGIIa-L1-ZOTU4 were more abundant in the ONSCS (Figure 2).

Nutrients (NO_3_^–^, NO_2_^–^, PO_4_^3–^, and SiO_3_^2–^) and temperature may have significantly (*P* < 0.05) shaped phytoplankton and MGII archaea community structures and potential activities between the PRP and ONSCS samples (Figure 2C). Meanwhile, the *Synechococcus*-ZOTU2, ZOTU4, *Cerataulina*-ZOTU9 may have shaped (*P* < 0.05) MGII archaea community structures and potential activities and the *Prochlorococcus*-ZOTU1, -ZOTU3, -ZOTU22 may have affected (*P* < 0.05) MGII archaea potential activities (Figure 2D).

Marine Group II and phytoplankton community structures have similar (Pearson: *P* < 0.05) changes in the PRP-PA-rRNA, PRP-FL-rRNA, and ONSCS-PA-rRNA genes (Supplementary Figure 6). The positive relationships between MGII and phytoplankton ZOTUs were closer for the PRP-FL-rRNA gene (Pearson: *R* = 0.90, *P* < 0.05) than for the PRP-PA-rRNA gene (Pearson: *R* = 0.72, *P* < 0.05); and these positive relationships were closer for the ONSCS-PA-rRNA gene (Pearson: *R* = 0.49, *P* < 0.05) than for the ONSCS-FL-rRNA gene (Pearson: *P* > 0.05) (Supplementary Figure 6). MGII and phytoplankton potential activities only had positive relationships in PRP-PA-rRNA (Pearson: *R* = 0.90, *P* < 0.05) and PRP-FL-rRNA (Pearson: *R* = 0.90, *P* < 0.05) except in the ONSCS (Pearson: *P* > 0.05) (Supplementary Figure 7).

**FIGURE 3:**
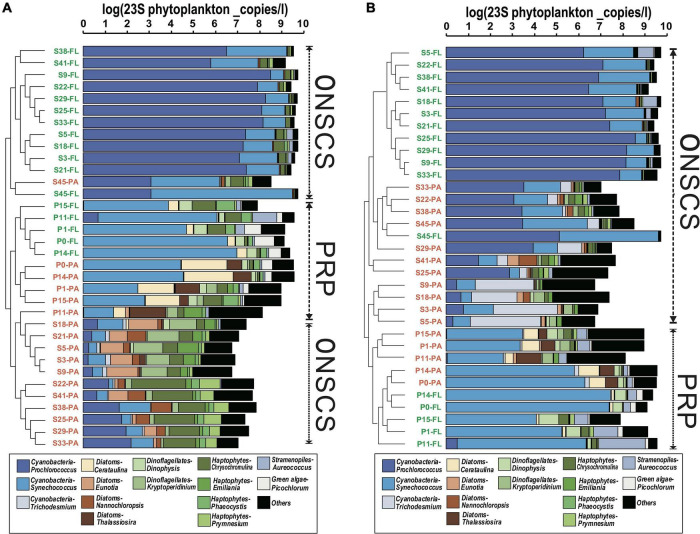
Cluster analysis of phytoplankton communities (genus level) based on the proportions in rRNA gene **(A)** and rRNA (cDNA, **B**) fractions. The total absolute abundances of phytoplankton (based on qPCR data) were shown at the top. Orange labels represented the PA fraction, and green labels represented the FL fraction.

In the PRP area, cyanobacteria-*Synechococcus* (average 33.8% in the rRNA gene and 46.7% in rRNA, respectively), diatoms-*Cerataulina*, and diatoms-*Thalassiosira* were dominant abundant/activity phytoplankton in PA fractions, and the cyanobacteria-*Synechococcus* (average 60.6% in the rRNA gene and 66.1% in rRNA, respectively), haptophytes-*Chrysochromulina*, green algae-*Picochlorum*, and dinoflagellates-*Dinophysis* were the main phytoplankton in FL fractions (Figure 3). In the ONSCS area, the cyanobacteria-*Prochlorococcus* (average 16.5% in the rRNA gene and 28.9% in rRNA, respectively), haptophytes-*Chrysochromulina*, cyanobacteria-*Synechococcus* (average 12.1% in the rRNA gene and 16.8% in rRNA, respectively), dinoflagellates-*Kryptoperidinium*, diatoms-*Eunotia*, diatoms-*Nannochloropsis*, and haptophytes-*Prymnesium* were prevalent phytoplankton in PA fractions, and the cyanobacteria-*Prochlorococcus* (average 74.2% in the rRNA gene and 75.2% in rRNA, respectively) and cyanobacteria-*Synechococcus* (average 18.9% in the rRNA gene and 18.7% in rRNA, respectively) were abundant in FL fractions (Figure 3).

**FIGURE 4:**
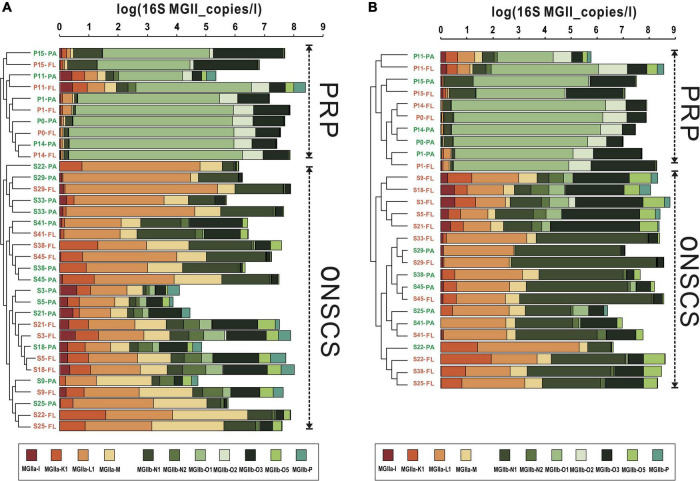
Cluster analysis of MGII communities (genus level) based on the proportions in rRNA gene **(A)** and rRNA (cDNA, **B**) fractions. Warm colors represent the MGIIa subgroups, and cool colors represent the MGIIb subgroups. The total absolute abundances of MGII (based on qPCR data) were shown at the top. Orange labels represent the PA fraction, and green labels represent the FL fraction.

The main MGII genera in rRNA gene and rRNA fractions were illustrated in Figures 4A,B, respectively; the samples could be divided into the PRP and ONSCS areas based on the proportion of the MGII genus. In the PRP area, MGIIb (-O1, -O3, -O2, and -N1) were the most abundant/activity MGII in PA (average 90.5% in rRNA gene and 92.3% in rRNA, respectively) and FL (average 92.7% in rRNA gene and 94.7% in rRNA, respectively) fractions. In the ONSCS area, the MGIIa (-L1, -M, and -K1) and MGIIb (-N1 and -O3) were prevalent in PA (average 67.8% in the rRNA gene and 51.1% in rRNA, respectively) and FL (average 60.2% in the rRNA gene and 37.2% in rRNA, respectively) fractions.

### Correlations Between Phytoplankton and Marine Group II Archaeal Zero-Radius Operational Taxonomic Units and Environmental Parameters

**FIGURE 5:**
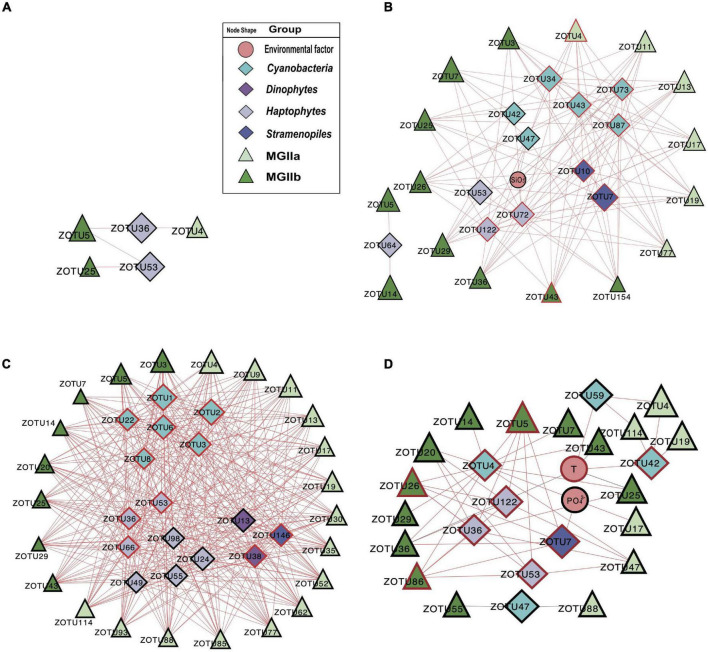
Network analysis showing significant correlations (Pearson: *R*^2^ > 0.64; *P* < 0.01) in phytoplankton and MGII ZOTU absolute abundances (ZOTUs proportion multiplied by total qPCR abundance) and environmental variables (including salinity, temperature, NO_3_^–^, NH_4_^+^, SiO_3_^2–^, and PO_4_^3–^) in the PRP-PA-rRNA gene fraction (**A**, *n* = 5), the PRP-FL-rRNA gene fraction (**B**, *n* = 5), the ONSCS-PA-rRNA gene fraction (**C**, *n* = 12), and the ONSCS-FL-rRNA gene fraction (**D**, *n* = 12). Red lines indicate positive relationships and gray lines negative relationships. The size of each node shows the ZOTUs’ absolute abundances. The hub ZOTUs were marked with red borders.

**FIGURE 6:**
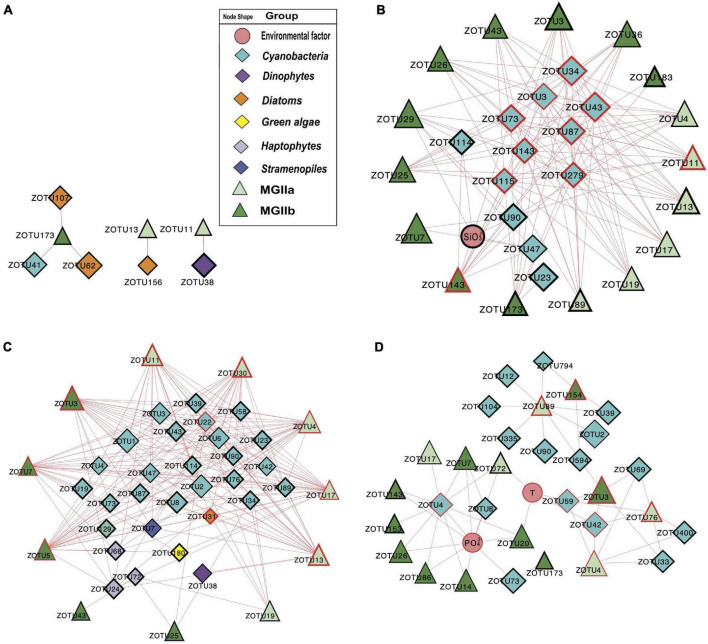
Network analysis showing significant correlations (Pearson: *R*^2^ > 0.64; *P* < 0.01) in rRNA (cDNA) abundances of phytoplankton and MGII ZOTUs (ZOTU proportion multiplied by total qPCR abundance) and environmental variables (including salinity, temperature, NO_3_^–^, NH_4_^+^, SiO_3_^2–^, and PO_4_^3–^) in the PRP-PA-rRNA fraction (**A**, *n* = 5), PRP-FL-rRNA fraction (**B**, *n* = 5), ONSCS-PA-rRNA fraction (**C**, *n* = 6), and ONSCS-FL-rRNA fraction (**D**, *n* = 12). Red lines indicate positive relationships and gray lines negative relationships. The size of each node shows the ZOTUs’ absolute abundance. The hub ZOTUs were marked with red borders.

To investigate the potential impact of phototrophs on the distributions of those MGII in the PRP and ONSCS areas, the absolute abundance of phytoplankton and MGII ZOTUs fractions were used to build the PRP-PA-rRNA gene, PRP-FL-rRNA gene, ONSCS-PA-rRNA gene, and ONSCS-FL-rRNA gene networks after filtering the low-abundance ZOTUs (*R*^2^ > 0.64 and *P* < 0.01; Figure 5). The rRNA (cDNA) abundance of phytoplankton and MGII ZOTUs were used to build the PRP-PA-rRNA, PRP-FL-rRNA, ONSCS-PA-rRNA, and ONSCS-FL-rRNA networks (*R*^2^ > 0.64; *P* < 0.01; Figure 6).

In the “PRP-PA-rRNA gene” network, only one negative and three positive relationships were found between three MGII (represented 12.7% of total MGII reads) and two phytoplankton ZOTUs (2.3% of total phytoplankton reads) (Figure 5A). The abundant MGIIb-O3-ZOTU5 constituting 11.4% of total MGII reads was positively correlated with a haptophytes-*Chrysochromulina* (ZOTU35) and negatively correlated to a haptophyte (ZOTU53) in abundance. In the “PRP-FL-rRNA gene” network, a total of 111 positive relationships were found between 16 MGII ZOTUs (69.4% of total MGII reads) and 12 phytoplankton ZOTUs (18.5% of total phytoplankton reads) (Figure 5B). Both MGIIa and MGIIb ZOTUs were included in this network with diverse cyanobacteria, haptophytes, and stramenopiles. High-proportion MGIIb (65.8% of total MGII reads) and minor MGIIa (3.6% of total MGII reads) ZOTUs had positive relationships with multiple phytoplankton. The most abundant MGII MGIIb-O1-ZOTU7 constituting 30.8% of total MGII reads was positively correlated with four cyanobacteria-*Synechococcus* ZOTUs, one haptophytes-*Chrysochromulina* ZOTU, and one haptophytes-*Phaeocystis* ZOTUs (Figure 5B). There were miscellaneous ZOTUs identified as hubs in the PRP-FL-rRNA gene network (analysis top 10 ZOTUs), which may play important roles and shape the MGII community, including one MGIIa-L1 ZOTU, one MGIIb-N1 ZOTU, two diatoms’ ZOTUs, three haptophytes’ ZOTUs, four cyanobacterial ZOTUs, and one stramenopile’s ZOTU (Figure 5B).

The difference in network complexity was also found in the RNA samples of the PRP area between the FL and the PA fractions (Figure 6A). Specifically, only five positive correlations were found in the PRP-PA-rRNA network, while 120 positive correlations were found in the PRP-FL-rRNA one. Both the PRP-PA-rRNA gene (taking 12.7% of MGII) and PRP-PA-rRNA (taking 2.4% of MGII) networks demonstrated that only low proportions of MGII had positive relationships with phytoplankton (Figures 5A, 6A). While in the PRP-FL-rRNA gene and PRP-FL-rRNA networks, there were high proportions of MGII positively connecting with phytoplankton (cyanobacteria, haptophytes, and stramenopiles). Especially, the abundant MGIIb-O1-ZOTU7 (taking 30.8 and 33.7% of total MGII reads in the PRP-FL-rRNA gene and PRP-FL-rRNA, respectively) was positively connected with diverse phytoplankton in the PRP-FL-rRNA gene network, but it was only positively connected with cyanobacteria-*Synechococcus* in the PRP-FL-rRNA network (Figures 5B, 6B). There were one MGIIa-M ZOTU, one MGIIb-O1 ZOTU, and eight cyanobacterial ZOTUs identified as hub ZOTUs in the PRP-FL-rRNA network (Figure 6B).

In the “ONSCS-PA-rRNA gene” network, a total of 271 positive relationships were found between 24 MGII ZOTUs (represented 71.4% of total MGII reads) and 18 phytoplankton ZOTUs (27.9% of total phytoplankton reads; Figure 5C). The high proportion of MGIIa (50.9% of total MGII reads) and relatively lower MGIIb (20.5% of total MGII reads) were positively connected with phytoplankton. Cyanobacteria, diatoms, green algae, haptophytes, and stramenopiles had positive relationships with mainly MGIIa ZOTUs MGIIa-L1-ZOTU4 (12.5% of total MGII reads), -L1-ZOTU9 (10.3% of total MGII reads), and -M-ZOTU11 (11.7% of total MGII reads) in this network. In the ONSCS-FL-rRNA gene fraction, a total of 38 positive relationships were found between 17 MGII ZOTUs (represented 27.4% of total MGII reads) and 8 phytoplankton ZOTUs (2.3% of total phytoplankton reads) (Figure 5D). Similar proportions of MGIIa (14.7% of total MGII reads) and MGIIb ZOTUs (12.6% of total MGII reads) were found to have positive relationships with phytoplankton. Four cyanobacterial ZOTUs, two dinoflagellates ZOTUs, one stramenopile’s ZOTU, and one haptophyte’s ZOTU were identified as the top 10 hub ZOTUs in the ONSCS-PA-rRNA gene network (Figure 5C).

Unlike the networks in the PRP area, the ONSCS-PA-rRNA network was more complex than the ONSCS-FL-rRNA network (Figures 6C,D). In the ONSCS-PA-rRNA network (Figure 6C), 11 MGII ZOTUs (70.5% of MGII) and 29 phytoplankton ZOTUs (49.3% of phytoplankton) had 279 positive correlations. Both of MGIIa (taking 28.2% of MGII) and MGIIb (42.3% of MGII) had high proportions that positively correlated with cyanobacteria, diatoms, haptophytes, and stramenopiles, such as the abundant MGIIa-L1-ZOTU4 (12.9% of MGII) and MGIIb-N1-ZOTU7 (34.9% of MGII). In the ONSCS-FL-rRNA network (Figure 6D), 15 MGII ZOTUs (87.8% of MGII) and 16 cyanobacterial ZOTUs (10.7% of phytoplankton) had 35 positive correlations. Compared to MGIIa (13.4% of MGII), the higher active abundance of MGIIb (34.2% of MGII) was positively correlated with phytoplankton. The dominating MGII ZOTU MGIIb-N1-ZOTU3 (28.8% of MGII) and MGIIa-L1-ZOTU4 (11.3% of MGII) had positive relationships with cyanobacteria. Five MGIIa ZOTUs, three MGIIb ZOTUs, one cyanobacterial ZOTU, and one diatom ZOTU were identified as the top 10 hub ZOTUs in the ONSCS-PA-rRNA network (Figure 6C), while three cyanobacterial ZOTUs in the ONSCS-FL-rRNA network was identified as the top 10 hub ZOTUs (Figure 6D).

## Discussion

### Factors Affecting the Abundance and Activity of Marine Group II Archaea in the Pearl River Plume and Open Regions of the Northern South China Sea Areas

The higher abundances of phytoplankton and MGII in the PRP than in the ONSCS area and the concentrations of nutrients were significantly higher in the PRP than in the ONSCS area, which might result from the high nutrient input from the river and estuary (Supplementary Figures 3, 4; Xie et al., 2018). Most PRP and ONSCS sites had higher MGII abundance in FL than the PA fraction (Supplementary Figure 4 and Supplementary Table 6, except in S45 and P0 sites), which might be due to the higher abundance of phytoplankton in FL than the PA fraction (Supplementary Figures 4, 5). This is different from the observation that MGII archaea prefer to attach to particles in the estuarine and coastal regions (Xie et al., 2018; Orellana et al., 2019).

The significant positive correlations (Supplementary Figure 5) between the abundance of MGII and phytoplankton in both PA and FL in the PRP and ONSCS surface waters support the notion that phytoplankton may have an important control on the MGII distribution (Galand et al., 2010; Orsi et al., 2015; Xie et al., 2018). As indicated by many genomic studies (Martin-Cuadrado et al., 2015; Zhang et al., 2015; Orsi et al., 2016; Rinke et al., 2019; Tully, 2019; Damashek et al., 2021), MGII archaea have metabolic potentials to attach to phytoplankton and utilize phytoplankton-derived organic matter. However, evidence is lacking that the phytoplankton would have to couple with MGII archaea for their growth.

The potential MGII activity based on RNA analysis has been poorly reported (Zhang et al., 2015; Liu et al., 2017; Xie et al., 2018). The MGII rRNA had a significantly higher abundance than the rRNA gene in PA and FL fractions (Supplementary Figure 4), indicating that the MGII cells were active. The MGII rRNA abundance was higher in FL than the PA fraction in most waters (Supplementary Table 6), suggesting that MGII tend to live in an FL lifestyle. In particular, the PA MGII have stronger correlations to phytoplankton than the FL MGII in the PRP and ONSCS (Supplementary Figure 5), which may be caused by increased capacity for surface adhesion, transcriptional regulation, and high molecular catabolism in the MGII genomes in PA fraction (Orsi et al., 2015). Stronger positive correlations of MGII archaeal and phytoplankton abundances were found in rRNA than in rRNA genes, suggesting that the growth of phytoplankton might stimulate the activity of the heterotrophic MGII archaea (Beier et al., 2015; Damashek et al., 2021).

### Dispersal Limitation May Play an Important Role in Surface Marine Group II Assembly

MGIIa and MGIIb were found to partition in different niches and have varying metabolic characteristics, which indicate that the subgroups of MGII may represent distinct ecotypes (Iverson et al., 2012; Zhang et al., 2015; Xie et al., 2018; Rinke et al., 2019; Santoro et al., 2019; Tully, 2019; Dai et al., 2020). Here, we found that the stochastic processes of dispersal limitation may be the most important factor in MGII community assembly (see Supplementary Material). Dispersal limitation means that the movement of MGII (colonization) in a new location is restricted (Zhou and Ning, 2017). This is revealed in the NMDS and cluster analysis of MGII communities (Figures 2C,D, 4A,B). Samples of the PRP and ONSCS can be well distinguished based on MGII and phytoplankton ZOTUs compositions. Multiple *Synechococcus* and MGIIb ZOTUs had similar trends in rRNA gene and rRNA samples and were more abundant in the PRP than in the ONSCS, while *Prochlorococcus* and MGIIa were more abundant in the ONSCS (Figures 2, 3). *Synechococcus* are much more abundant in nutrient-rich regions than in oligotrophic areas, but *Prochlorococcus* are the most abundant photosynthetic prokaryotes in the oligotrophic oceans (Partensky et al., 1999). Picocyanobacteria are major primary producers in the world’s oceans (Li, 1994) and contribute to the marine DOM and particulate organic matter (POM) pool in the surface ocean (Jiao et al., 2011; Gontikaki et al., 2013), indicating that *Synechococcus* and *Prochlorococcus* were important speciation drivers of MGII communities in the PRP and ONSCS.

The MGIIb genera are well adapted to environmental conditions in the PRP that is characterized by high concentrations of remineralized DOM from river inputs and phytoplankton bloom (Chen et al., 2004; Sieczko et al., 2015; He et al., 2020). Generally, MGIIb MAGs encoded a higher fraction of peptidases and membrane transporters compared to MGIIa (Orellana et al., 2019) and the MGIIb proportions increased in nutrient-enriched waters during winter (Galand et al., 2010; Orellana et al., 2019). The MGIIa genera (included: -L1, -M, and -K1) represented most of MGII in ONSCS. The MGIIb genera (included: -O1, -O2, and -O3) were the majority of MGII in the PRP (Figures 4A,B), which had relatively higher nutrients than the ONSCS (NO_3_^–^, NO_2_^–^, PO_4_^3–^, and SiO_3_^2–^; Supplementary Table 3), suggesting that MGIIb distribution may respond to high nutrient availability (Figure 2C). MGIIb encode a large number of peptidases and membrane transporters in their genomes, suggestive of the capacity for DOM and POM degradation (Galand et al., 2010; Orellana et al., 2019; Dai et al., 2020), which may help to maintain their ecological dominancy in the PRP.

### Correlations Between Phytoplankton and Marine Group II Archaeal Zero-Radius Operational Taxonomic Units in the Pearl River Plume and Open Regions of the Northern South China Sea Areas

The network analyses were used to explore the potential interactions for phytoplankton and MGII archaea in PA and FL fractions from the near estuarine environment (PRP) to open oceans (ONSCS). An advantage of the absolute phytoplankton and MGII ZOTU abundances is that it may lead to less spurious correlations (Friedman and Alm, 2012; Berry and Widder, 2014).

Most of the interactions between phytoplankton and MGII archaea were positive relationships in all networks. This may be caused by the special metabolic potentials of MGII archaea, which can obtain and utilize the phytoplankton-derived organic carbon (Martin-Cuadrado et al., 2015; Zhang et al., 2015; Orsi et al., 2016; Rinke et al., 2019; Tully, 2019; Damashek et al., 2021). Similar patterns are found in rRNA genes and rRNA-based networks that the PRP-FL and ONSCS-PA networks were much larger and more complex than the PRP-PA and ONSCS-FL networks (Figures 5, 6). In the DOM/nutrient-rich PRP area (near estuary) (He et al., 2020), more positive relationships of MGII and phytoplankton were found in FL than the PA fraction, in which MGII ZOTUs consisted of almost MGIIb, whereas in the DOM/nutrient-poor ONSCS area, more complex positive relationships of MGII and phytoplankton were found in PA than the FL fraction, consisting of over 50% MGIIa ZOTUs in the ONSCS-PA-rRNA gene network (Figure 5). This is coincident with the difference in genome characters that MGIIb genera have a greater capacity for DOM degradation and all MGIIa genera possess the archaeal flagella gene operon playing the function for cell adhesion (Rinke et al., 2019). The metabolic characteristic difference of MGII genera makes multiple MGIIb abundant in the nutrient-rich PRP area (Figure 4), and MGIIb tend to have an FL lifestyle that makes the networks in PRP-FL more complex than in the PRP-PA (Figures 5, 6).

The changes in phytoplankton populations and activities might cause the shift of the MGII population and activities between FL and PA fractions (Supplementary Figure 5). The dominant abundant/active phytoplankton were found in cyanobacteria, dinoflagellates, diatoms, green algae, and stramenopiles in the PRP and ONSCS areas (Figure 3). Multiple-hub ZOTUs in haptophytes, cyanobacteria, and stramenopiles indicate that the phytoplankton population was an important factor in the MGII distribution and activity in PRP-FL and ONSCS-PA networks (Figures 5, 6). The cyanobacteria were the only hub phytoplankton in the PRP-FL-rRNA and ONSCS-FL-rRNA networks, but some cyanobacteria with haptophytes consisted of the ONSCS-PA-rRNA network (Figure 6). The phytoplankton such as cyanobacteria and haptophytes can supply organic cofactors/siderophores; DMSP and glycolate (Malin, 2006; Uronen et al., 2007; Tsuji and Yoshida, 2017), which could be utilized by MGII, may influence the MGII communities and activities in the PRP and ONSCS areas.

Some MGII ZOTUs can live in both PA and FL lifestyles, which displayed similar relationships with phytoplankton in the PRP-FL-rRNA gene and ONSCS-PA-rRNA gene networks (Figure 5). For example, the abundant MGII ZOTUs MGIIa-L1-ZOTU4, MGIIa-M-ZOTU11, and MGIIb-N1-ZOTU3 were present in the PRP-FL-rRNA gene and ONSCS-PA-rRNA gene networks, which responded to various phytoplankton ZOTUs in cyanobacteria, haptophytes, and stramenopiles. The MGIIa-M may have the potential to attach to particular organic matter through the presence of archaeal flagellin and a high-affinity cytochrome *bd* oxidase in surrounding phytoplankton (Tambalo et al., 2010; Meshcheryakov and Wolf, 2016; Tully, 2019). MGIIa-L1 have flagella genes and are increased with the blooms of the diatom at Port Hacking in spring (Rinke et al., 2019). The MGIIb-N1 have no flagella genes but have thrombospondin and flotillin genes, which may make them form cell aggregation (Rinke et al., 2019). The MGIIa-L1 and MGIIb-N1 genera have the potential to shift between different lifestyles to better utilize phytoplankton-derived organic matters and adapt to the environmental changes, making them widely distributed in the PRP and ONSCS areas.

Although the published genomic data have revealed some unique metabolic potentials of MGII, the physiological and biochemical characteristics of MGII diverse subgroups are still unknown, which need the pure cultures/enrichments to unscramble. The observation of syntrophic interactions within MGII and phytoplankton in the coastal surface water may provide valuable information for future research on this mysterious group of organisms that still resist being brought into pure culture.

## Data Availability Statement

The datasets presented in this study can be found in online repositories. The names of the repository/repositories and accession number(s) can be found in the article/Supplementary Material.

## Author Contributions

SC, LF, and CZ developed the idea and designed the study. SC, JT, YC, and WW processed and analyzed the data. SC, LF, and CZ wrote the manuscript. All authors contributed to the article and approved the submitted version.

## Conflict of Interest

The authors declare that the research was conducted in the absence of any commercial or financial relationships that could be construed as a potential conflict of interest.

## Publisher’s Note

All claims expressed in this article are solely those of the authors and do not necessarily represent those of their affiliated organizations, or those of the publisher, the editors and the reviewers. Any product that may be evaluated in this article, or claim that may be made by its manufacturer, is not guaranteed or endorsed by the publisher.

## Footnotes

1
http://blast.ncbi.nlm.nih.gov

2
huttenhower.sph.harvard.edu/galaxy
