# Supplementary material for: Interactions Between Marine Group II Archaea and Phytoplankton Revealed by Population Correlations in the Northern Coast of South China Sea
Source: Front Microbiol. 2022 Jan 25;12:785532. doi: 10.3389/fmicb.2021.785532 (PMC8821943; doi:10.3389/fmicb.2021.785532)
Supplement: Supplementary file 1 [file Data_Sheet_1.docx]

**Supplementary Information**

**Supplementary text**

**Design and evaluation** **of new MGII primers**

The currently available primers GII-554F/Eury-806R targeting the 16S rRNA genes of MGII archaea were designed nearly twenty years ago (Massana et al., 1997; Teira et al., 2004) and only cover a limited subpopulation regarding to the substantial diversity of this order of archaea as demonstrated by recently studies (Galand et al., 2010; Rinke et al., 2019; Tully, 2019). Therefore, it is urgently demanded to design an updated set of primers.

To take the advantage of MGII archaea metagenome-assembled genomes (MAGs) recovered in the past years, we downloaded the 16S rRNA gene sequences of MGII archaea from the Genome Taxonomy Database (GTDB, bac-arc_ssu_r86, https://gtdb.ecogenomic.org) and those published by Tully et al (2019) and Galand et al (2010). Raw sequences were aligned to the SILVA database (SSURef_Nr99_132. SILVA_13_12_17) using default commend in Sina-1.2.11 (https://www.arb-silva.de). The aligned sequences were imported to ARB-6.0.6 and added to the existing SILVA tree (SSURef_Nr99_132. SILVA_13_12_17) in the ARB-6.0.6 package by using the ARB Parsimony (Quick add marked) function (Ludwig et al., 2004). A finalized alignment of 812 sequences was used to build a customized MGII archaea 16S rRNA gene database for primers design in ARB-6.0.6 (Supplementary Database_for_MGII.fa). We selected all potential primers using the following criteria: 1) length 18–25 bp; 2) had < 10% mismatches to MGII archaeal representative sequences, didn’t match bacteria, and only matched Thermoplasmata) reverse primer specific to MGII and no degenerate position; 4) AT/GC ratio of 40%–60%; 5) Tm 52–62 °C. The newly designed MGII 16S rRNA gene primers were Ar-559F: THTTATTGGGCCTAAAACGTCCG and MGII-771R: TATCTAATCCGGTTCGTGCCCCT.

The sensitivity and specificity of the newly designed primer set Ar-559F/MGII-771R targeting MGII archaeal 16S rRNA genes were verified in comparison to the GII-554F/Eury-806R set frequently used in previous studie (Massana et al., 1997; Teira et al., 2004). First, primer sequences were analyzed *in silico* by using Arb-6.0.6 with the customized MGII 16S rRNA gene database as reference. All primers adequately covered all genera of MGIIa and MGIIb archaea except primer Eury-806R, which missed sequences of MGIIb-Q2, a genus widely distribute in gulf stream, Antarctic province and deep water down to 5000 m (Supplementary Table S1) (Rinke et al., 2019). 34.1% of the hits of the primer Ar-559F were non-MGII archaea hits including Marine Benthic Group D/Deep Hydrothermal Vent Euryarchaeota, other Thermoplasmata and some other uncultured archaea lineages. No hits to bacterial sequences were detected by searching the online tool of SILVA (https://www.arb-silva.de/search/testprime) (Ludwig et al., 2004) to the SILVA138.1 database.

The amplification efficiency and specificity of primers on MGII archaea were tested on DNA samples from open regions of the northern South China Sea (ONSCS) (S5-PA, S9-PA, S22-PA, and S38-PA, Figure 1) and Pearl River estuary sampling trip on 2016.08 (B, C, E, and D, Supplementary Table S2). Coverages of primer sets to MGII archaea 16S rRNA genes in these samples were examined by conducting qPCR analysis and high-throughput sequencing. In general, more gene copies were detected in estuarine samples by the new Ar-559F/MGII-771R primer set (Supplementary Table S3, Figures S1). Similar abundances of MGII archaea, however, were found by both the new primer set and the commonly used primer set GII-554F/Eury-806R in ONSCS samples. The archaeal V4-V5 primers Arch524F10extF/Arch958RmodR were used to collect longer MGII sequences from the same site to check the primer coverage using Check Primer (Simple e-PCR) in TBtools (Chen et al., 2020). The results showed a higher coverage of total MGII reads by the Ar-559F/MGII-771R primers (97.3%) than the GII-554F/Eury-806R primers (85.9%). The main MGII ZOTUs can only be quantified by the newly designed MGII primers (Supplementary Figure S2b with red dots). The high-throughput sequencing of new Ar-559F/MGII-771R primer set was then used for specificity test. On average, 99.3% of the obtained sequences belonged to MGII (Supplementary Table S4). Exception occurred at the estuarine site B, which gave 3.4% non-specific amplifications.

In summary, by comparing to the old primers, the newly designed primer set Ar-559F/MGII-771R shows better coverage to the MGIIb-Q2 genus and estuarine populations and sufficient specificity. Therefore, these new primers can be adequately used in quantification of MGII archaea in global oceans.

**Ecological process determination in iCAMP**

The iCAMP has been widely used in ecological process determination, which has high accuracy, precision, sensitivity, and specificity on simulated communities (Dong et al., 2021; García-Palacios et al., 2021; Stopnisek and Shade, 2021; Sun et al., 2021; Zhou and Ning, 2017). Both deterministic and stochastic processes operate simultaneously in the assembly of local communities and embedded in different ecological processes, including selection, dispersal, speciation or diversification, and ecological drift (Chase, 2010; Chase and Myers, 2011; Ofiteru et al., 2010; Zhou and Ning, 2017).

Based on 16S rRNA gene sequence and environmental variable analysis, the dispersal limitation was the most important ecological process in the community assembly of MGII in both PA and FL fractions (48.4%–54.3%), followed by the drift ﻿and others (35.3%–40.4%) in NSCS areas (Supplementary Figures S8a and S8b). The drift and others here, including ecological drift, stochastic diversification, weak selection, and/or weak dispersal. The "iCAMP" has a lower performance in the competition predominates selection, environmental filtering, and biotic interactions, which might underestimate selection when difference is not detectable (Ning et al., 2020). Thus, the further network approaches disentangle biotic interactions from abiotic filtering and capture special MGII selection.

**REFERENCES**

Chase, J. M. (2010). Stochastic community assembly causes higher biodiversity in more productive environments. *Science*. 328, 1388–1391. doi:10.1126/science.1187820.

Chase, J. M., and Myers, J. A. (2011). Disentangling the importance of ecological niches from stochastic processes across scales. *Philos. Trans. R. Soc. B Biol. Sci.* 366, 2351–2363. doi:10.1098/rstb.2011.0063.

Chen, C., Chen, H., Zhang, Y., Thomas, H. R., Frank, M. H., He, Y., et al. (2020). TBtools: an integrative toolkit developed for interactive analyses of big biological data. *Mol. Plant* 13, 1194–1202. doi:10.1016/j.molp.2020.06.009.

Dong, Y., Sanford, R. A., Connor, L., Chee-sanford, J., Wimmer, B. T., Iranmanesh, A., et al. (2021). Differential structure and functional gene response to geochemistry associated with the suspended and attached shallow aquifer microbiomes from the Illinois Basin, IL. *Water Res*. 202, 117431. doi:10.1016/j.watres.2021.117431.

Galand, P. E., Gutiérrez-Provecho, C., Massana, R., Gasol, J. M., and Casamayor, E. O. (2010). Inter-annual recurrence of archaeal assemblages in the coastal NW Mediterranean Sea (Blanes Bay Microbial Observatory). *Limnol. Oceanogr.* 55, 2117–2125. doi:10.4319/lo.2010.55.5.2117.

García-Palacios, P., Crowther, T. W., Dacal, M., Hartley, I. P., Reinsch, S., Rinnan, R., et al. (2021). Evidence for large microbial-mediated losses of soil carbon under anthropogenic warming. *Nat. Rev. Earth Environ*. 2, 507–517. doi:10.1038/s43017-021-00178-4.

Ludwig, W., Strunk, O., Westram, R., Richter, L., Meier, H., Yadhukumar., et al. (2004). ARB: a software environment for sequence data. *Nucleic Acids Res*, 32(4), 1363-1371.

Massana, R., Murray, A. E., Preston, C. M., and DeLong, E. F. (1997). Vertical distribution and phylogenetic characterization of marine planktonic Archaea in the Santa Barbara Channel. *Appl. Environ. Microbiol*. 63, 50–56. doi:10.1128/AEM.63.1.50-56.1997.

Ning, D., Yuan, M., Wu, L., Zhang, Y., Guo, X., Zhou, X., et al. (2020). A quantitative framework reveals ecological drivers of grassland microbial community assembly in response to warming. *Nat. Commun*. 11. doi:10.1038/s41467-020-18560-z.

Ofiteru, I. D., Lunn, M., Curtis, T. P., Wells, G. F., Criddle, C. S., Francis, C. A., et al. (2010). Combined niche and neutral effects in a microbial wastewater treatment community. *Proc. Natl. Acad. Sci.* 107, 15345–15350. doi:10.1073/pnas.1000604107.

Rinke, C., Rubino, F., Messer, L. F., Youssef, N., Parks, D. H., Chuvochina, M., et al. (2019). A phylogenomic and ecological analysis of the globally abundant Marine Group II archaea (Ca. Poseidoniales ord. nov.). *ISME J*. 13, 663–675. doi:10.1038/s41396-018-0282-y.

Stopnisek, N., and Shade, A. (2021). Persistent microbiome members in the common bean rhizosphere: an integrated analysis of space, time, and plant genotype. *ISME J*. doi:10.1038/s41396-021-00955-5.

Sun, C., Zhang, B., Ning, D., Zhang, Y., Dai, T., Wu, L., et al. (2021). Seasonal dynamics of the microbial community in two full-scale wastewater treatment plants: Diversity, composition, phylogenetic group based assembly and co-occurrence pattern. *Water Res*. 200, 117295. doi:10.1016/j.watres.2021.117295.

Teira, E., Reinthaler, T., Pernthaler, A., Pernthaler, J., and Herndl, G. J. (2004). Combining catalyzed reporter deposition-fluorescence in situ hybridization and microautoradiography to detect substrate utilization by bacteria and archaea in the deep ocean. *Appl. Environ. Microbiol*. 70, 4411–4414. doi:10.1128/AEM.70.7.4411-4414.2004.

Tully, B. J. (2019). Metabolic diversity within the globally abundant Marine Group II Euryarchaea offers insight into ecological patterns. *Nat. Commun.* 10, 271. doi:10.1038/s41467-018-07840-4.

Zhou, J., and Ning, D. (2017). Stochastic community assembly: does it matter in microbial ecology? *Microbiol. Mol. Biol*. *Rev*. 81, 1–32. doi:10.1128/mmbr.00002-17.

**Supplementary Tables**

Table S1. *In silico* analysis of the sensitivity and specificity of primer sets Ar-559F/MGII-771R and GII-554F/Eury-806R.

|  | Sequences | Ar-559F | MGII-771R | GII-554F | Eury-806R | Ar-559F/ MGII-771R | GII-554F/ Eury-806R |
| --- | --- | --- | --- | --- | --- | --- | --- |
| MGII | 811 | 743(91.6%) | 736(90.8%) | 704(86.8%) | 732(90.3%) | 710(87.7%) | 681(84.0%) |
| MGIIa | 208 | 179(86.1%) | 171(82.2%) | 151(72.6%) | 154(74.0%) | 166(79.8%) | 160(76.9%) |
| MGIIb | 603 | 564(93.5%) | 565(93.7%) | 553(91.7%) | 578(95.9%) | 544(90.2%) | 521(86.4%) |
| MGIIb-Q2 | 28 | 24(85.7%) | 27(96.4%) | 28(100.0%) | 0(0.0%) | 21(75.0%) | 0(0.0%) |
| not-MGII archaea | 2569 | 875(34.1%) | 3(0.1%) | 20(0.8%) | 3(0.1%) | 1(0.0%) | 0(0.0%) |

Table S2. Location and salinities of extra Pearl River estuarine sites.

| Samples | Longitude | Salinity |
| --- | --- | --- |
|  | and Latitude | (psu) |
| B | 113.72257E 23.63474N | 1.17 |
| C | 113.98833E 22.36417N | 11.24 |
| E | 113.76166E 22.17444N | 22.21 |
| D | 113.78472E 22.05056N | 28.05 |

Table S3. Quantification of 16S MGII rRNA genes in real seawater samples by qPCR analysis with primer sets Ar-559F/MGII-771R and GII-554F/Eury-806R. qPCR was conducted in triplicates for each sample.

| Samples | Ar-559F/ MGII-771R (copies/μl DNA) | | GII-554F/ Eury-806R (copies/μl DNA) | |
| --- | --- | --- | --- | --- |
|  | Mean | STDEV | Mean | STDEV |
| B | 14438.34 | 3483.47 | 4147.41 | 1114.87 |
| C | 43063.31 | 19136.00 | 25462.16 | 8435.92 |
| E | 38431.76 | 13513.45 | 21608.68 | 7303.86 |
| D | 197369.78 | 59087.90 | 95165.41 | 28715.13 |
| S5-PA | 207.00 | 99.55 | 230.00 | 46.21 |
| S9-PA | 83.70 | 50.93 | 48.70 | 6.71 |
| S22-PA | 12719.66 | 3234.04 | 12939.05 | 3212.66 |
| S38-PA | 21954.82 | 2328.84 | 20488.75 | 4079.99 |

Table S4. The specificity of the Ar-559F/MGII-771R primer pair in seawater samples.

| Groups | B | C | E | D | S5-PA | S9-PA | S22-PA | S38-PA | Average |
| --- | --- | --- | --- | --- | --- | --- | --- | --- | --- |
| MGII | 96.6% | 99.8% | 99.0% | 100.0% | 100.0% | 98.9% | 100.0% | 100.0% | 99.3% |
| Methanobacteriales | 0.9% | 0.0% | 0.8% | 0.0% | 0.0% | 0.3% | 0.0% | 0.0% | 0.3% |
| Bacteria/unclassified | 2.4% | 0.2% | 0.1% | 0.0% | 0.0% | 0.8% | 0.0% | 0.0% | 0.5% |

Table S5. Location and physicochemical parameters of sampling sites.

| Sites | Regions | Longitude  and Latitude | Temperature | Salinity | NH_4_^+^ | NO_3_^-^ | NO_2_^-^ | PO_4_^3-^ | SiO_3_^2-^ |
| --- | --- | --- | --- | --- | --- | --- | --- | --- | --- |
|  |  |  | (°C) | (psu) | (μmol/l) | (μmol/l) | (μmol/l) | (μmol/l) | (μmol/l) |
| P0 | PRP^*^ | 22.1315N 113.8055E | 27.00 | 28.00 | 0.12 | 1.20 | 1.02 | 0.17 | 6.22 |
| P1 |  | 22.16829N 114.76995E | 27.30 | 33.50 | 1.21 | 0.87 | 0.11 | 0.19 | 4.62 |
| P11 |  | 20.93074N 114.53560E | 27.70 | 33.80 | 0.00 | 1.05 | 0.17 | 0.04 | 2.77 |
| P14 |  | 21.73028N 114.06397E | 26.90 | 33.60 | 0.00 | 1.10 | 0.93 | 0.11 | 6.12 |
| P15 |  | 21.48938N 113.27994E | 27.50 | 33.50 | 0.00 | 1.44 | 0.60 | 0.17 | 6.91 |
|  |  |  |  |  |  |  |  |  |  |
| S3 | ONSCS^**^ | 21.58882N 115.09203E | 28.20 | 33.80 | 0.29 | 0.68 | 0.00 | 0.03 | 1.84 |
| S5 |  | 20.95563N 115.45126E | 28.00 | 33.90 | 1.84 | 0.59 | 0.00 | 0.03 | 1.79 |
| S9 |  | 20.34189N 114.88400E | 28.20 | 33.70 | 0.00 | 0.64 | 0.00 | 0.01 | 1.56 |
| S18 |  | 20.56542N 113.79810E | 28.10 | 33.70 | 0.00 | 0.83 | 0.00 | 0.01 | 1.81 |
| S21 |  | 19.66232N 114.63783E | 28.20 | 33.50 | 0.06 | 0.74 | 0.00 | 0.00 | 1.74 |
| S22 |  | 18.84920N 111.84779E | 28.80 | 33.20 | 0.00 | 0.63 | 0.00 | 0.00 | 2.04 |
| S25 |  | 19.05533N 111.48941E | 29.30 | 33.80 | 0.40 | 0.71 | 0.00 | 0.00 | 1.82 |
| S29 |  | 19.32779N 111.01020E | 30.20 | 33.60 | 0.58 | 0.45 | 0.00 | 0.00 | 1.04 |
| S33 |  | 18.59601N 111.09649E | 30.00 | 33.70 | 0.98 | 0.87 | 0.00 | 0.00 | 1.56 |
| S38 |  | 17.50298N 110.79919E | 28.80 | 33.70 | 0.00 | 0.51 | 0.00 | 0.00 | 1.65 |
| S41 |  | 17.87598N 110.57100E | 29.80 | 33.50 | 0.00 | 0.82 | 0.00 | 0.00 | 1.78 |
| S45 |  | 18.35548N 110.24998E | 30.20 | 33.50 | 0.06 | 0.74 | 0.00 | 0.00 | 2.38 |

^*^PRP = Pearl River Plume.

^**^ONSCS = Open regions of Northern South China Sea.

Table S6. Abundances (copies/l) of phytoplankton and MGII rRNA sequences in PRP (n = 5) and ONSCS (n = 12) samples.

| Sites | Regions | 23S phytoplankton rRNA gene | 23S phytoplankton rRNA gene | 23S phytoplankton rRNA | 23S phytoplankton rRNA | 16S MGII rRNA gene | 16S MGII rRNA gene | 16S MGII rRNA | 16S MGII rRNA |
| --- | --- | --- | --- | --- | --- | --- | --- | --- | --- |
|  |  | in PA | in FL | in PA | in FL | in PA | in FL | in PA | in FL |
| P0 | PRP^*^ | 3.46E+09 | 1.25E+09 | 4.84E+08 | 2.04E+09 | 4.81E+07 | 3.41E+07 | 9.76E+06 | 7.55E+07 |
| P1 |  | 9.09E+08 | 1.34E+09 | 2.21E+09 | 3.45E+09 | 1.46E+07 | 7.18E+07 | 5.02E+07 | 1.84E+08 |
| P11 |  | 1.35E+08 | 3.59E+09 | 3.90E+08 | 5.67E+09 | 2.20E+05 | 2.48E+08 | 5.89E+05 | 3.59E+08 |
| P14 |  | 3.73E+09 | 2.30E+09 | 1.80E+09 | 1.94E+09 | 2.63E+07 | 7.24E+07 | 2.95E+07 | 7.85E+07 |
| P15 |  | 9.24E+08 | 8.11E+07 | 7.31E+08 | 1.71E+08 | 4.96E+07 | 6.38E+06 | 3.20E+07 | 1.15E+07 |
|  |  |  |  |  |  |  |  |  |  |
| S3 | ONSCS^**^ | 7.81E+06 | 3.89E+09 | 5.67E+06 | 1.21E+10 | 1.25E+04 | 7.87E+07 | 8.36E+04 | 6.11E+08 |
| S5 |  | 5.64E+06 | 5.36E+09 | 4.03E+06 | 7.03E+09 | 7.38E+03 | 5.26E+07 | 2.29E+04 | 2.60E+08 |
| S9 |  | 5.46E+06 | 5.37E+09 | 2.01E+06 | 6.18E+09 | 5.42E+04 | 4.36E+07 | 2.27E+04 | 2.06E+08 |
| S18 |  | 2.37E+07 | 5.34E+09 | 3.00E+06 | 1.70E+09 | 7.20E+04 | 1.03E+08 | 3.23E+04 | 1.12E+08 |
| S21 |  | 1.13E+07 | 2.59E+09 | 2.44E+07 | 7.41E+09 | 2.84E+04 | 3.25E+07 | 2.67E+04 | 2.39E+08 |
| S22 |  | 5.40E+07 | 2.58E+09 | 1.04E+08 | 7.53E+09 | 1.27E+06 | 7.46E+07 | 4.17E+06 | 3.97E+08 |
| S25 |  | 2.21E+07 | 4.24E+09 | 2.06E+07 | 7.52E+09 | 5.71E+05 | 3.88E+07 | 2.46E+06 | 1.97E+08 |
| S29 |  | 3.11E+07 | 5.04E+09 | 1.60E+08 | 1.73E+10 | 1.71E+06 | 7.64E+07 | 1.17E+07 | 3.59E+08 |
| S33 |  | 1.04E+07 | 3.59E+09 | 4.05E+07 | 1.22E+10 | 4.97E+05 | 4.57E+07 | 1.35E+06 | 2.45E+08 |
| S38 |  | 7.13E+07 | 3.47E+09 | 5.11E+08 | 2.03E+10 | 2.20E+06 | 3.78E+07 | 4.46E+07 | 2.91E+08 |
| S41 |  | 4.82E+07 | 1.45E+09 | 2.88E+08 | 1.01E+10 | 2.73E+06 | 2.74E+06 | 9.18E+06 | 5.70E+07 |
| S45 |  | 3.22E+08 | 5.52E+09 | 1.12E+09 | 1.03E+10 | 3.06E+07 | 1.68E+07 | 1.53E+08 | 3.61E+08 |

^*^PRP = Pearl River Plume.

^**^ONSCS = Open regions of Northern South China Sea.

Table S7. Properties of phytoplankton 23S rRNA gene sequencing data: Reads, coverage and normalized Shannon index.

| **Samples** | **rRNA gene** | | | | **rRNA** | | | |
| --- | --- | --- | --- | --- | --- | --- | --- | --- |
|  | **ID** | **Reads** | **Coverage** | **Shannon** | **ID** | **Reads** | **Coverage** | **Shannon** |
| P0-PA | D1 | 10908 | 99.4% | 2.86 | C1 | 14772 | 99.6% | 3.03 |
| P0-FL | D2 | 10118 | 99.4% | 2.29 | C2 | 12733 | 99.6% | 2.39 |
| P1-PA | D5 | 13354 | 99.3% | 3.78 | C5 | 12305 | 99.3% | 4.23 |
| P1-FL | D6 | 7098 | 99.1% | 3.13 | C6 | 11769 | 99.4% | 3.22 |
| S3-PA | D7 | 7494 | 99.5% | 4.44 | C7 | 11456 | 99.7% | 3.25 |
| S3-FL | D8 | 6338 | 98.4% | 2.02 | C8 | 8220 | 99.2% | 1.94 |
| S5-PA | D9 | 8058 | 99.7% | 4.19 | C9 | 11198 | 99.9% | 3.68 |
| S5-FL | D10 | 4665 | 98.2% | 1.92 | C10 | 4703 | 98.6% | 2.21 |
| S9-PA | D11 | 15367 | 99.9% | 4.17 | C11 | 11308 | 99.9% | 3.44 |
| S9-FL | D12 | 6303 | 98.5% | 1.45 | C12 | 6227 | 99.0% | 1.60 |
| P11-PA | D15 | 15270 | 99.3% | 3.94 | C15 | 5567 | 98.4% | 4.26 |
| P11-FL | D16 | 7414 | 98.9% | 3.13 | C16 | 5239 | 98.5% | 2.84 |
| P14-PA | D19 | 7551 | 99.1% | 2.80 | C19 | 7334 | 99.1% | 3.25 |
| P14-FL | D20 | 5918 | 99.3% | 2.20 | C20 | 9185 | 99.3% | 2.37 |
| P15-PA | D21 | 9660 | 99.0% | 3.62 | C21 | 7698 | 99.0% | 4.14 |
| P15-FL | D22 | 7280 | 99.0% | 3.20 | C22 | 7893 | 99.4% | 3.37 |
| S18-PA | D25 | 10142 | 98.6% | 4.09 | C25 | 5662 | 99.1% | 3.84 |
| S18-FL | D26 | 6853 | 98.4% | 2.02 | C26 | 6183 | 98.4% | 1.97 |
| S21-PA | D29 | 8899 | 99.0% | 4.18 | - | - | - | - |
| S21-FL | D30 | 4083 | 98.3% | 1.77 | C30 | 6303 | 98.9% | 1.87 |
| S22-PA | D33 | 9869 | 99.3% | 4.96 | C33 | 5091 | 99.1% | 3.37 |
| S22-FL | D34 | 3803 | 98.1% | 1.61 | C34 | 3825 | 98.7% | 1.87 |
| S25-PA | D37 | 7348 | 98.7% | 4.47 | C37 | 3914 | 97.8% | 3.67 |
| S25-FL | D38 | 5628 | 98.9% | 1.54 | C38 | 3317 | 98.0% | 1.39 |
| S29-PA | D41 | 13770 | 99.4% | 4.28 | C41 | 6420 | 98.5% | 2.84 |
| S29-FL | D42 | 7401 | 98.9% | 1.66 | C42 | 4597 | 99.0% | 1.59 |
| S33-PA | D45 | 13861 | 99.9% | 4.24 | C45 | 5616 | 99.7% | 3.05 |
| S33-FL | D46 | 5358 | 98.8% | 1.59 | C46 | 3581 | 98.7% | 1.81 |
| S38-PA | D47 | 11387 | 99.4% | 4.20 | C47 | 5621 | 98.5% | 3.31 |
| S38-FL | D48 | 7730 | 99.2% | 1.89 | C48 | 6421 | 99.3% | 1.84 |
| S41-PA | D53 | 6755 | 98.4% | 4.75 | C53 | 7843 | 99.0% | 3.95 |
| S41-FL | D54 | 6470 | 98.6% | 2.55 | C54 | 4616 | 98.6% | 2.12 |
| S45-PA | D59 | 7146 | 98.3% | 3.07 | C59 | 7591 | 98.7% | 2.85 |
| S45-FL | D60 | 12099 | 99.5% | 1.94 | C60 | 7150 | 99.5% | 2.08 |

Table S8. Properties of archaeal 16S rRNA gene sequencing data: Reads, coverage and normalized MGII Shannon index.

| **Samples** | **rRNA gene** | | | | | | **rRNA** | | | |
| --- | --- | --- | --- | --- | --- | --- | --- | --- | --- | --- |
|  | **ID** | **Reads** | | **Coverage** | **MGII-Shannon** | | **ID** | **Reads** | **Coverage** | **MGII-Shannon** |
| P0-PA | D1 | | 11582 | 98.9% | | 2.55 | C1 | 14365 | 99.3% | 2.18 |
| P0-FL | D2 | | 49366 | 99.7% | | 2.45 | C2 | 30263 | 99.6% | 2.39 |
| P1-PA | D5 | | 8100 | 98.1% | | 2.45 | C5 | 14137 | 99.1% | 2.15 |
| P1-FL | D6 | | 35564 | 99.6% | | 2.44 | C6 | 18689 | 99.2% | 2.29 |
| S3-PA | D7 | | 21297 | 99.5% | | 2.83 | C7 | 17890 | 100.0% | - |
| S3-FL | D8 | | 33324 | 99.3% | | 3.62 | C8 | 18320 | 98.6% | 3.21 |
| S5-PA | D9 | | 11003 | 99.4% | | 3.07 | C9 | 13848 | 100.0% | - |
| S5-FL | D10 | | 28953 | 99.2% | | 3.57 | C10 | 36440 | 99.3% | 3.00 |
| S9-PA | D11 | | 15677 | 99.6% | | 2.40 | C11 | 13754 | 100.0% | - |
| S9-FL | D12 | | 37538 | 99.5% | | 3.37 | C12 | 31344 | 99.4% | 3.10 |
| P11-PA | D15 | | 19240 | 98.7% | | 3.77 | C15 | 37912 | 99.7% | 2.75 |
| P11-FL | D16 | | 36850 | 99.4% | | 3.56 | C16 | 43146 | 99.4% | 3.34 |
| P14-PA | D19 | | 27997 | 99.5% | | 2.54 | C19 | 54986 | 99.8% | 2.25 |
| P14-FL | D20 | | 21602 | 99.4% | | 2.49 | C20 | 42272 | 99.7% | 2.28 |
| P15-PA | D21 | | 49514 | 99.6% | | 2.69 | C21 | 43440 | 99.6% | 2.21 |
| P15-FL | D22 | | 29162 | 99.4% | | 2.56 | C22 | 47414 | 99.7% | 2.05 |
| S18-PA | D25 | | 37914 | 99.6% | | 3.55 | - | - | - | - |
| S18-FL | D26 | | 35793 | 99.3% | | 3.61 | C26 | 29145 | 99.1% | 3.45 |
| S21-PA | D29 | | 32114 | 99.7% | | 2.84 | C29 | 61383 | 100.0% | - |
| S21-FL | D30 | | 34741 | 99.4% | | 3.48 | C30 | 25795 | 99.0% | 2.83 |
| S22-PA | D33 | | 16877 | 99.5% | | 2.00 | C33 | 26056 | 99.9% | 1.61 |
| S22-FL | D34 | | 34993 | 99.5% | | 2.93 | C34 | 34491 | 99.5% | 2.83 |
| S25-PA | D37 | | 31299 | 99.6% | | 2.50 | C37 | 37758 | 99.9% | 2.23 |
| S25-FL | D38 | | 34989 | 99.6% | | 3.01 | C38 | 37438 | 99.5% | 2.58 |
| S29-PA | D41 | | 32849 | 99.7% | | 1.86 | C41 | 56469 | 99.9% | 1.29 |
| S29-FL | D42 | | 36690 | 99.6% | | 2.12 | C42 | 52394 | 99.8% | 1.24 |
| S33-PA | D45 | | 37222 | 99.8% | | 2.12 | C45 | 61658 | 100.0% | - |
| S33-FL | D46 | | 31952 | 99.6% | | 2.19 | C46 | 27965 | 99.4% | 1.72 |
| S38-PA | D47 | | 7762 | 98.1% | | 2.87 | C47 | 33829 | 99.7% | 2.30 |
| S38-FL | D48 | | 32028 | 99.5% | | 3.09 | C48 | 23035 | 99.3% | 2.71 |
| S41-PA | D53 | | 36305 | 99.5% | | 2.66 | C53 | 50342 | 99.9% | 1.89 |
| S41-FL | D54 | | 47973 | 99.6% | | 2.76 | C54 | 47867 | 99.7% | 2.15 |
| S45-PA | D59 | | 28366 | 99.5% | | 3.04 | C59 | 24191 | 99.1% | 2.28 |
| S45-FL | D60 | | 23668 | 99.4% | | 2.77 | C60 | 16642 | 99.1% | 1.90 |
| B | P10 | | 19610 | 99.8% | | - | - | - | - | - |
| C | P19 | | 30829 | 99.8% | | - | - | - | - | - |
| E | P25 | | 24120 | 99.4% | | - | - | - | - | - |
| D | P31 | | 13948 | 99.0% | | - | - | - | - | - |

**Supplementary Figures**

FIGURE S1. 16S MGII rRNA gene abundance determined using the published primers GII-554F/Eury-806R vs. the newly designed primers Ar-559F/MGII-771R primers) in PRE water samples. The orange dots represent the low-salinity estuarine samples and blue dots represent the high-salinity marine water samples.


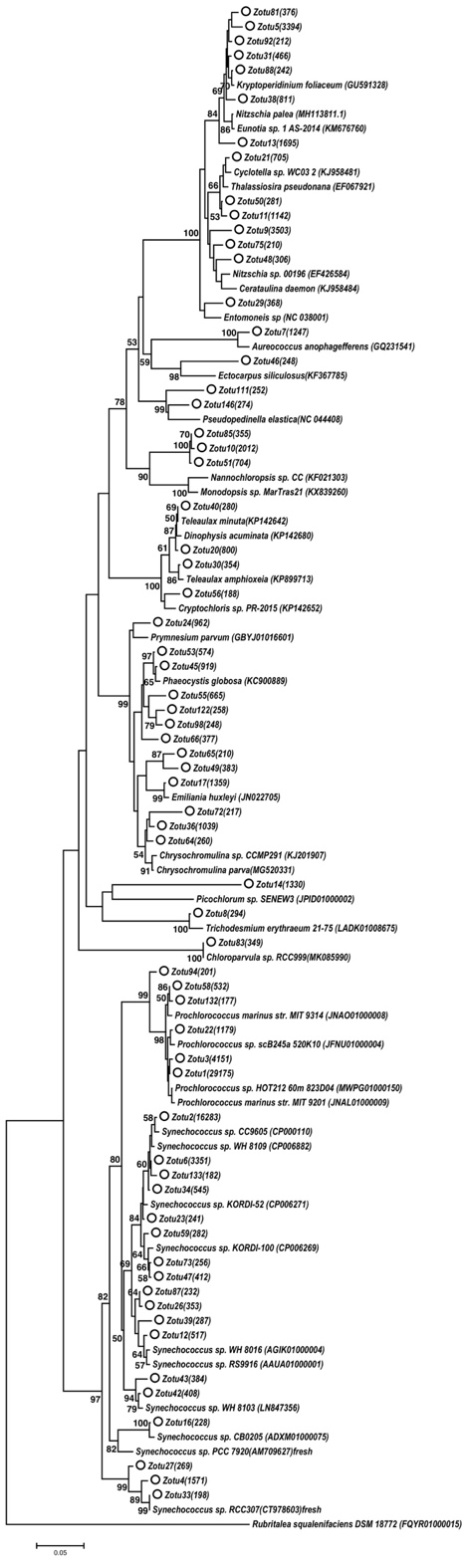

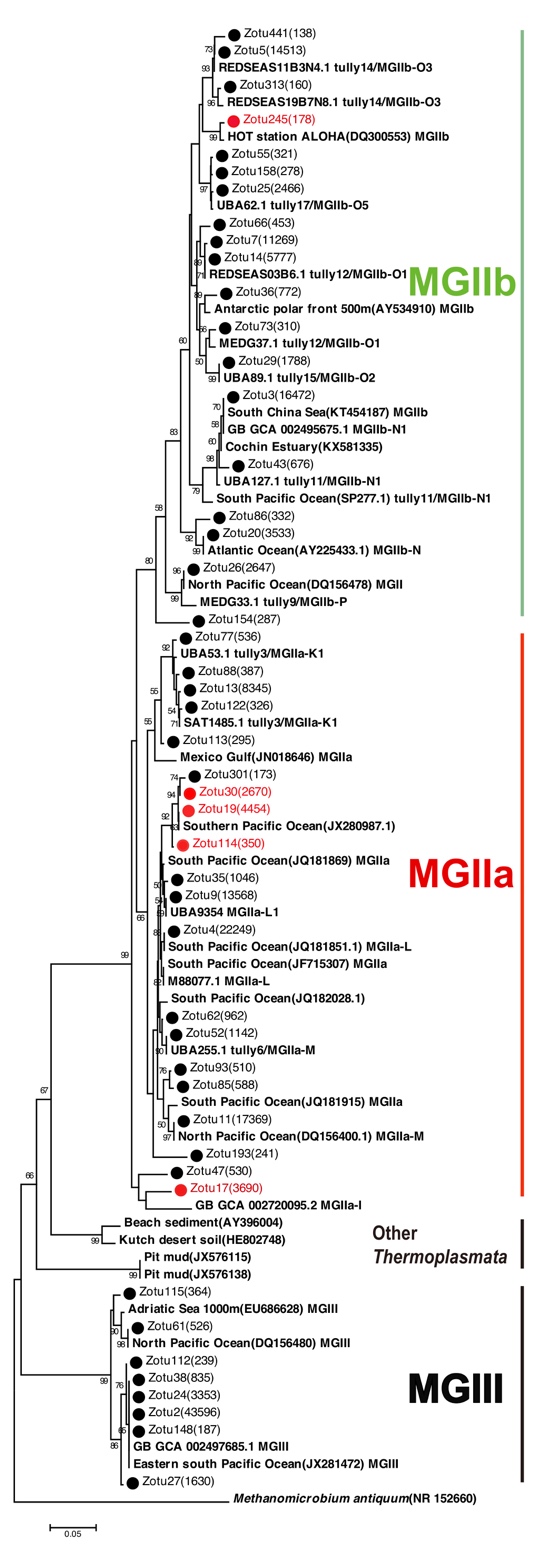


B)

A)

FIGURE S2. Neighbor-joining (NJ) phylogenetic tree of phytoplankton ZOTUs based on phytoplankton 23S rRNA gene sequences (A, the top 40 phytoplankton sequences either in PA or FL fraction); and the NJ phylogenetic tree of MGII ZOTUs based on archaeal 16S rRNA gene sequences (B, the top 40 MGII sequences either in PA or FL fraction). The red MGII ZOTUs can only be quantified by the newly designed MGII primers Ar-559F/ MGII-771R. Bootstrap values were both 1000 replicates.

FIGURE S3. Concentrations of nutrients (NH_4_^+^, NO_3_^-^, NO_2_^-^, PO_4_^3-^, and SiO_3_^2-^) in the PRP (n = 5) and ONSCS (n = 12) areas. The whiskers represent 50% of the data (from 25% to 75%). The median and mean values are marked using a solid line and a cross symbol, respectively.


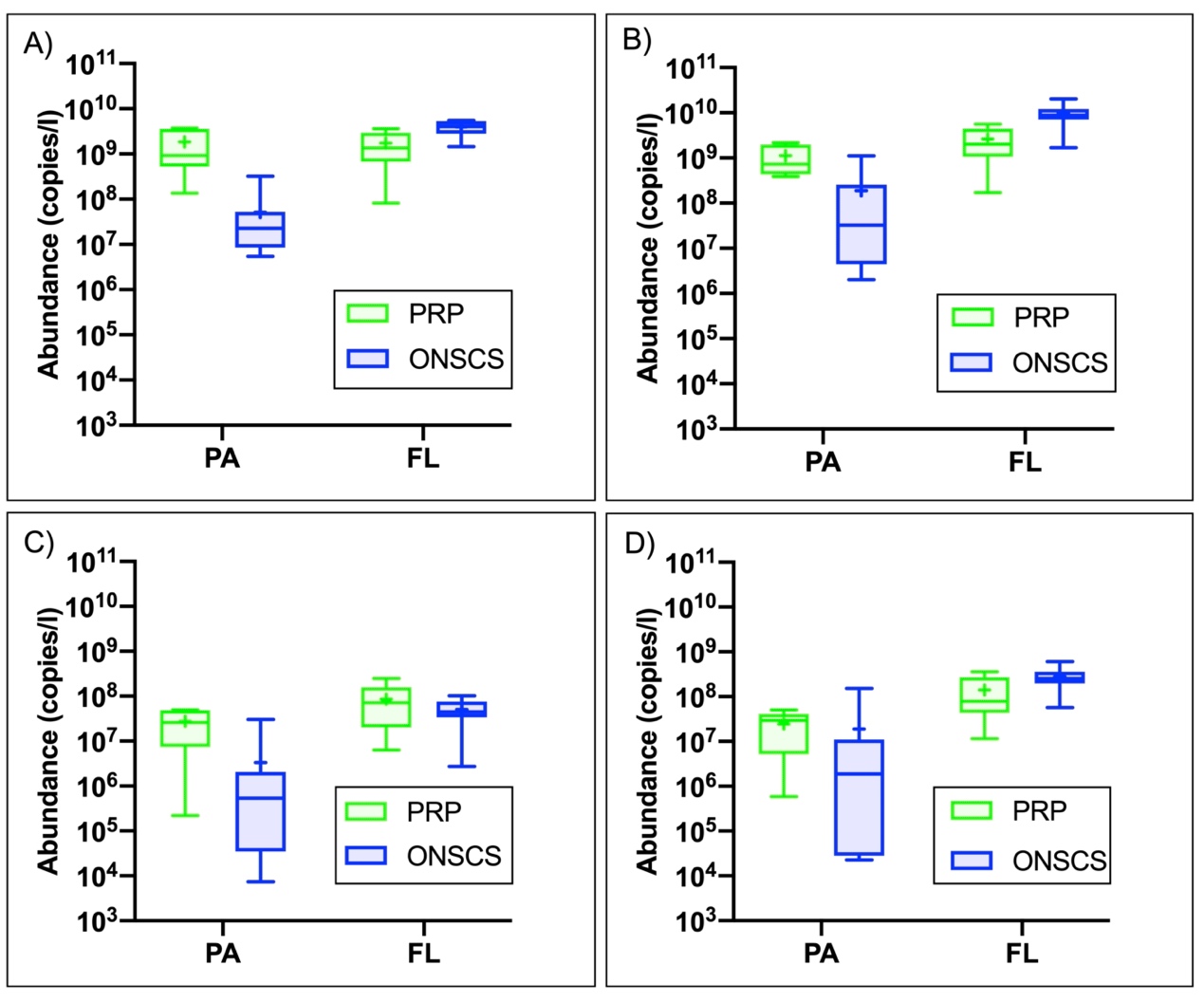
 FIGURE S4. Phytoplankton rRNA gene (a) and rRNA (b) abundances, and MGII rRNA gene (c) and rRNA (d) abundances in the PRP (n = 5) and ONSCS (n = 12) areas. The whiskers represent 50% of the data (from 25% to 75%). The median and mean values are marked using a solid line and a cross symbol, respectively.


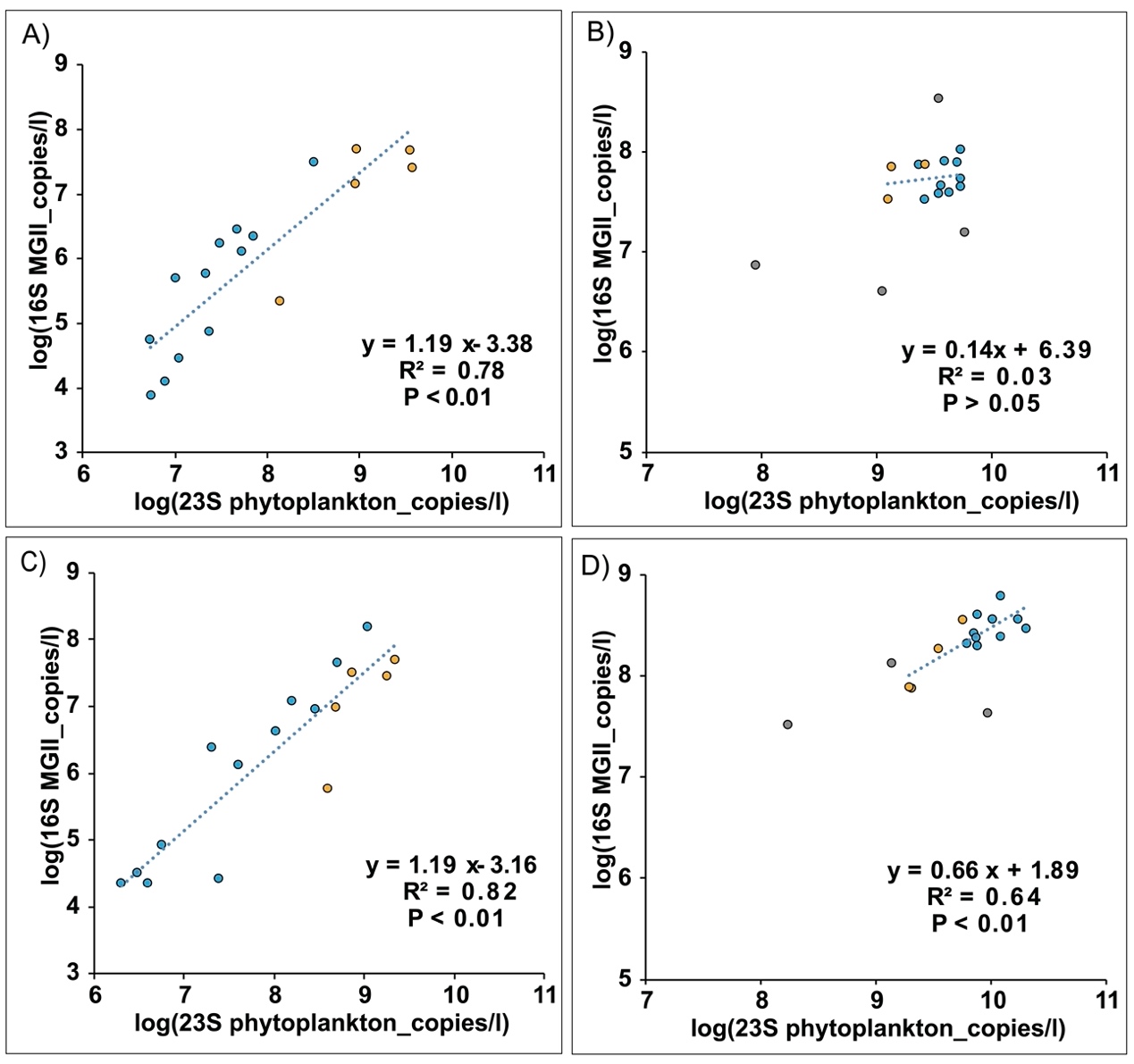


FIGURE S5. Pearson correlations in the abundances of phytoplankton and MGII for PA-rRNA gene (A, n = 17), FL-rRNA gene (B, n = 17), PA-rRNA (C, n = 13), and FL-rRNA (D, n = 14) fractions, respectively, in the study areas. Orange dots represent the PRP water samples and blue dots represent the ONSCS water samples. Gray dots represent the omitted samples (LOF > 1.5).


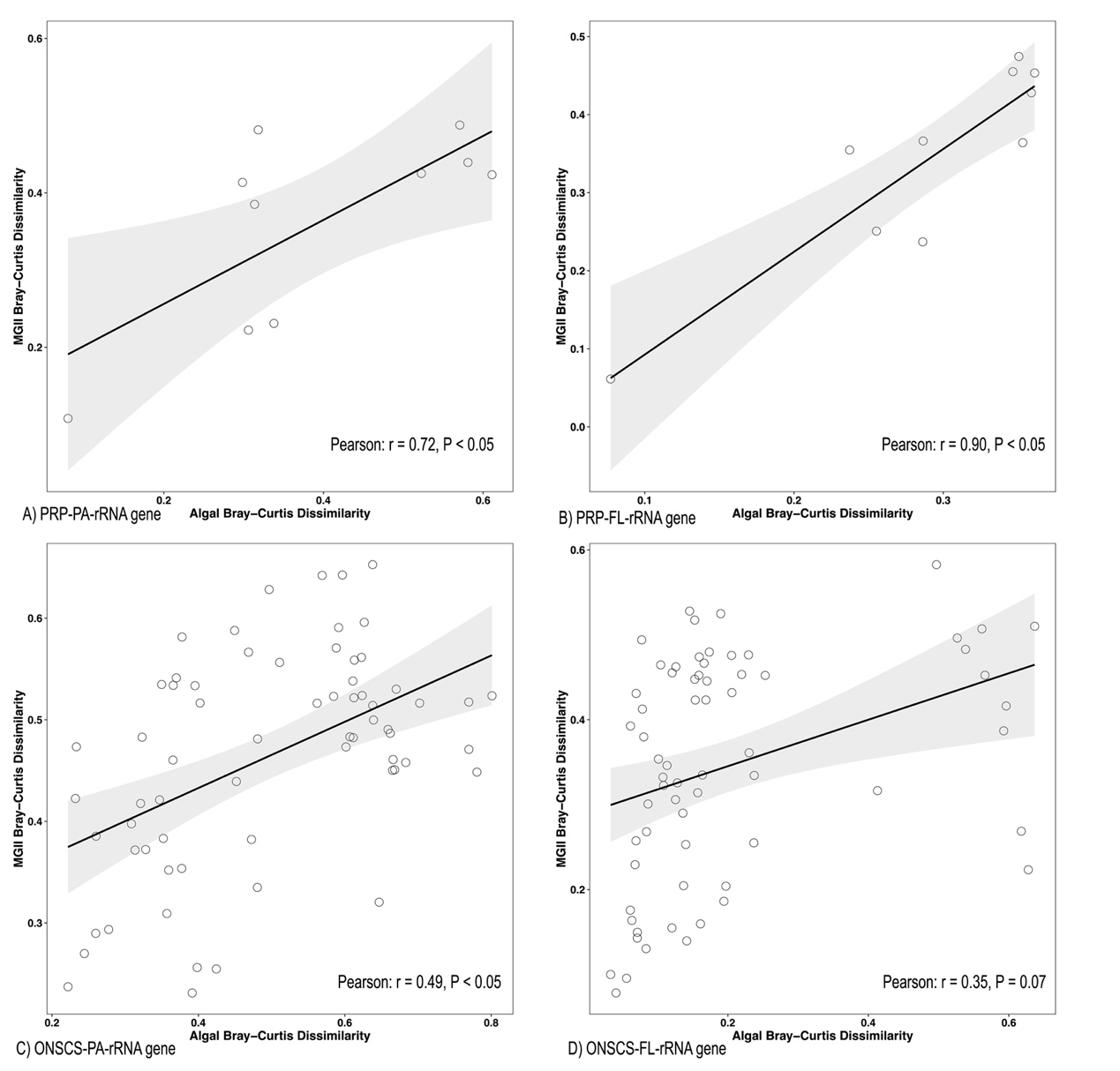


FIGURE S6. Mantel test of the phytoplankton community structure differences effects the MGII in PRP-PA-rRNA gene (A, n = 5), PRP-FL-rRNA gene (B, n = 5), ONSCS-PA-rRNA gene (C, n = 12), and ONSCS-FL-rRNA gene (D, n = 12) fractions. Based on Bray-Curtis similarity matrix (Pearson, permutation = 999) at the ZOTU level.


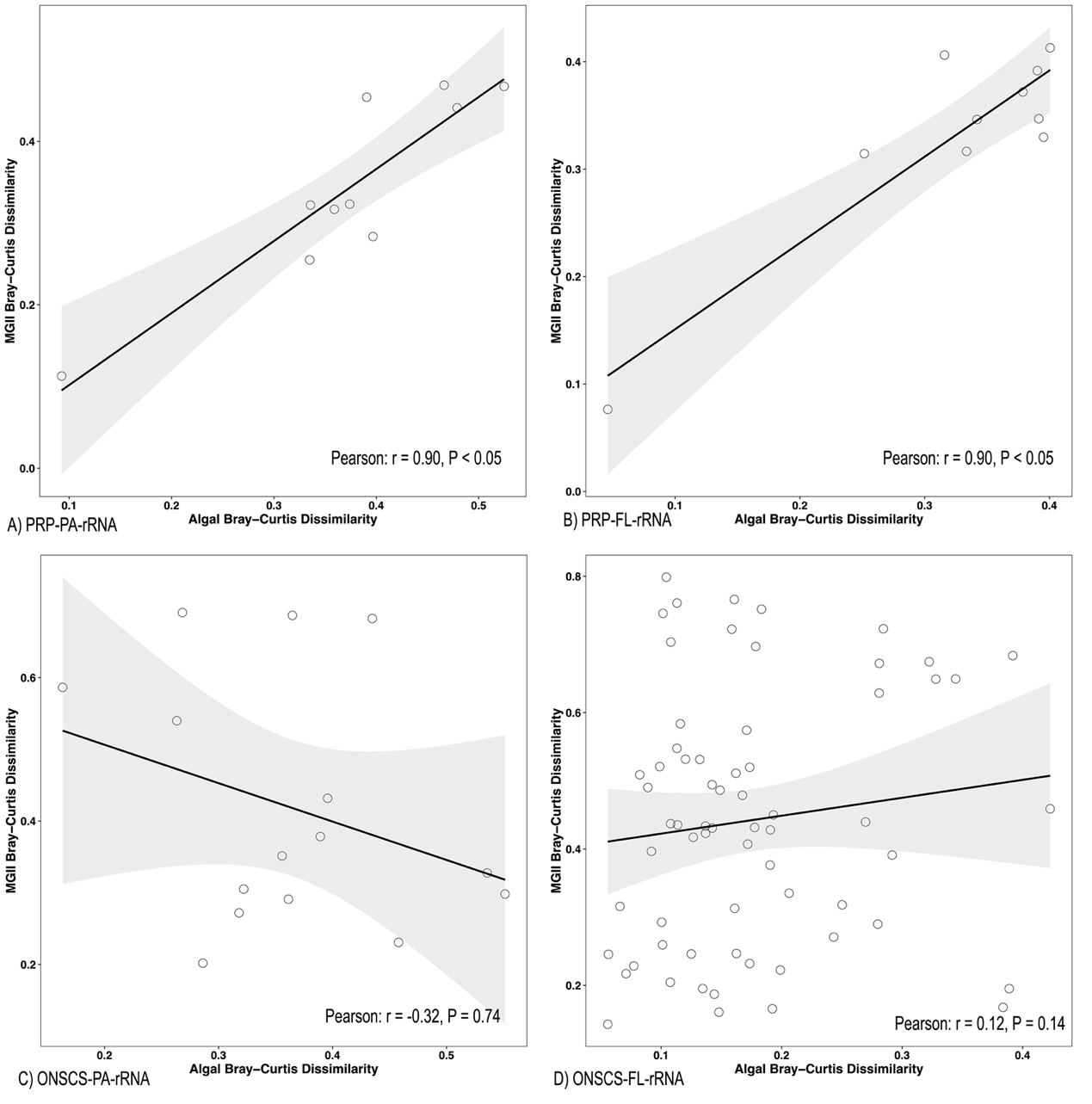


FIGURE S7. Mantel test of the phytoplankton potential activities differences effects the MGII in PRP-PA-rRNA (A, n = 5), PRP-FL-rRNA (B, n = 5), ONSCS-PA-rRNA (C, n = 6), and ONSCS-FL-rRNA (D, n = 12) fractions. Based on Bray-Curtis similarity matrix (Pearson, permutation = 999) at the ZOTU level.


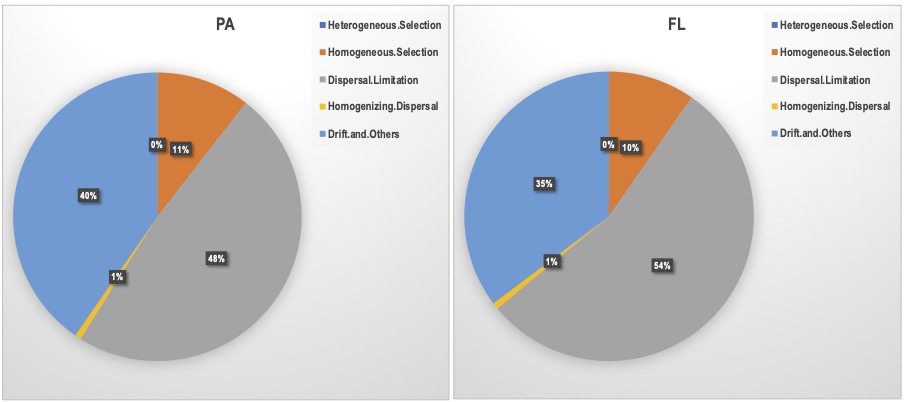


B)

A)

FIGURE S8. The performances of iCAMP (n = 1530, 34 biologically independent samples in each of 17 situations) estimated and expected relative importance of different individual ecological processes in PA (A) and FL fractions (B).
